# BMP9 reduces age-related bone loss in mice by inhibiting osteoblast senescence through Smad1-Stat1-P21 axis

**DOI:** 10.1038/s41420-022-01048-8

**Published:** 2022-05-06

**Authors:** Jing-zun Xu, Yan-man Zhou, Lin-lin Zhang, Xiao-jing Chen, Yu-ying Yang, Deng Zhang, Ke-cheng Zhu, Xiao-ke Kong, Li-hao Sun, Bei Tao, Hong-yan Zhao, Jian-min Liu

**Affiliations:** 1 Department of Endocrine and Metabolic Diseases, Shanghai Institute of Endocrine and Metabolic Diseases, Ruijin Hospital, Shanghai Jiao Tong University School of Medicine, Shanghai, China; 2 Shanghai National Clinical Research Center for Metabolic Diseases, Key Laboratory for Endocrine and Metabolic Diseases of the National Health Commission of the PR China, Shanghai Key Laboratory for Endocrine Tumor, State Key Laboratory of Medical Genomics, Ruijin Hospital, Shanghai Jiao Tong University School of Medicine, Shanghai, China; 3 Department of Nephrology, Provincial Hospital affiliated to Shandong First Medical University, Jinan, Shan Dong Province China

**Keywords:** Osteoporosis, Ageing

## Abstract

Age-related osteoporosis is characterized by the accumulation of senescent osteoblastic cells in bone microenvironment and significantly reduced osteogenic differentiation. Clearing of the senescent cells is helpful to improve bone formation in aged mice. Bone morphogenetic protein 9 (BMP9), a multifunctional protein produced and secreted by liver, was reported to improve osteoporosis caused by estrogen withdrawal. However, the mechanism of BMP9 has not been fully elucidated, and its effect on senile osteoporosis has not been reported. This study reveals that BMP9 significantly increases bone mass and improves bone biomechanical properties in aged mice. Furthermore, BMP9 reduces expression of senescent genes in bone microenvironment, accompanied by decreased senescence-associated secretory phenotypes (SASPs) such as Ccl5, Mmp9, Hmgb1, Nfkb1, and Vcam1. In vitro, Bmp9 treatment inhibits osteoblast senescence through activating Smad1, which suppresses the transcriptional activity of Stat1, thereby inhibits P21 expression and SASPs production. Furthermore, inhibiting the Smad1 signal in vivo can reverse the inhibitory effect of BMP9 on Stat1 and downstream senescent genes, which eliminates the protection of BMP9 on age-related osteoporosis. These findings highlight the critical role of BMP9 on reducing age-related bone loss by inhibiting osteoblast senescence through Smad1-Stat1-P21 axis.

## Abstract (web summary)

BMP9 inhibits cellular senescence by activation of Smad1, which suppresses the transcription of Stat1, resulting in decreased P21 expression and SASPs production in osteoblast. The anti-aging effect of BMP9 is benefit to improving age-related osteoporosis.

## Graphical abstract

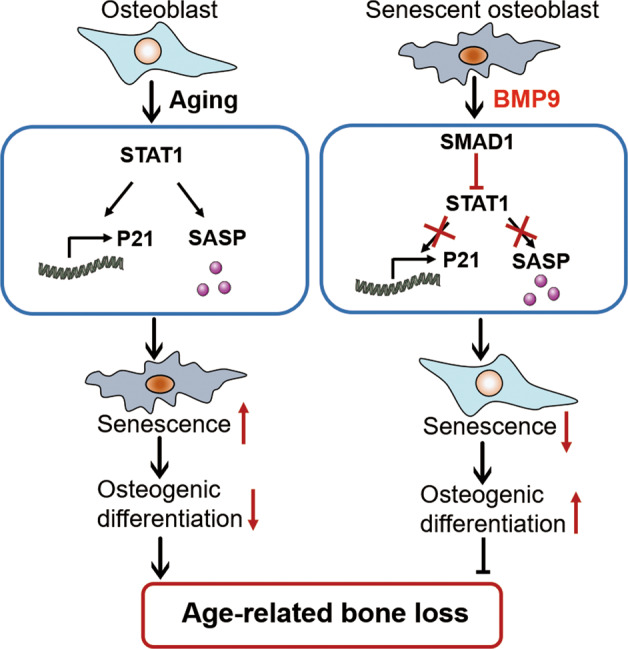

## Introduction

Aging is manifested as time-dependent degeneration of physiological functions at cellular, tissue, and organismal levels, making the individual more vulnerable to various diseases, such as osteoporosis [1]. Unlike postmenopausal osteoporosis due to estrogen deficiency, which is characterized by hyperactivity of osteoclasts, the main pathological feature of age-related bone loss is decreased osteoblast functions and reduced bone formation [2]. Growing evidence demonstrates that the proportion of senescent cells in bone microenvironment increases with age, which contributes to the deteriorated osteogenic capacity of osteoblasts [3–5]. Furthermore, senescent osteoblastic lineage cells produce a senescence-associated secretory phenotype (SASP) signal that is communicated to neighboring cells in local bone microenvironment, resulting in excessive production and secretion of chemokines and pro-inflammatory factors, thereby creating a toxic microenvironment which contributes to age-related bone loss [6–8].

With the rising of aging population, the incidence of osteoporosis and fracture is increasing significantly [1], treatment targeting cellular senescence in both postmenopausal and age-related osteoporosis has become a new strategy [9, 10]. In recent studies, senescence of bone marrow-derived mesenchymal stem cells is involved in ovariectomized (OVX)-induced osteoporosis [11] and deletion of senescent gene P16 rescues bone loss of OVX-mice [12], which highlights the pivotal role of senescence in estrogen deficiency-induced osteoporosis. In addition, pioneer work demonstrates that eliminating senescent cells or suppressing SASPs is effective in promoting bone formation in aged mice [6, 13]. Most of the previous studies focused on searching for exogenous reagents to inhibit senescence, termed as senolytic or senomorphic according to their effects on clearing senescent cells or suppressing SASPs secretion, respectively [14–16]. Researches on identification of the intrinsic anti-aging proteins should not be ignored.

Bone morphogenetic protein 9 (BMP9), mainly produced by liver and circulates in bloodstream, is a pleiotropic cytokine that regulates proliferation and differentiation in various cells [17, 18]. BMP9 has been demonstrated effective on promoting osteoblast differentiation [19] and improving fracture healing [20] in estrogen deficiency-induced osteoporosis. Nevertheless, the effect of BMP9 on age-related bone loss has not been clarified and studies about the regulation of BMP9 on senescence in skeleton are quite limited [21]. Since the role of cellular senescence in the pathogenesis of osteoporosis cannot be underestimated, it is necessary to clarify the effect of BMP9 on this pathological process.

In this study, we attempted to investigate the influence of BMP9 on bone quality and senescent bone microenvironment in aged mice. The precise mechanism was assessed on senescent osteoblast by RNA sequencing analysis and rescue experiments using specific activator and inhibitor. Findings from our research may expand our understanding of the functionalities of BMP9 and provide new perspective in the treatment of age-related osteoporosis.

## Results

### Aged mice exhibit reduced bone mass and aging bone microenvironment

**Fig. 1:**
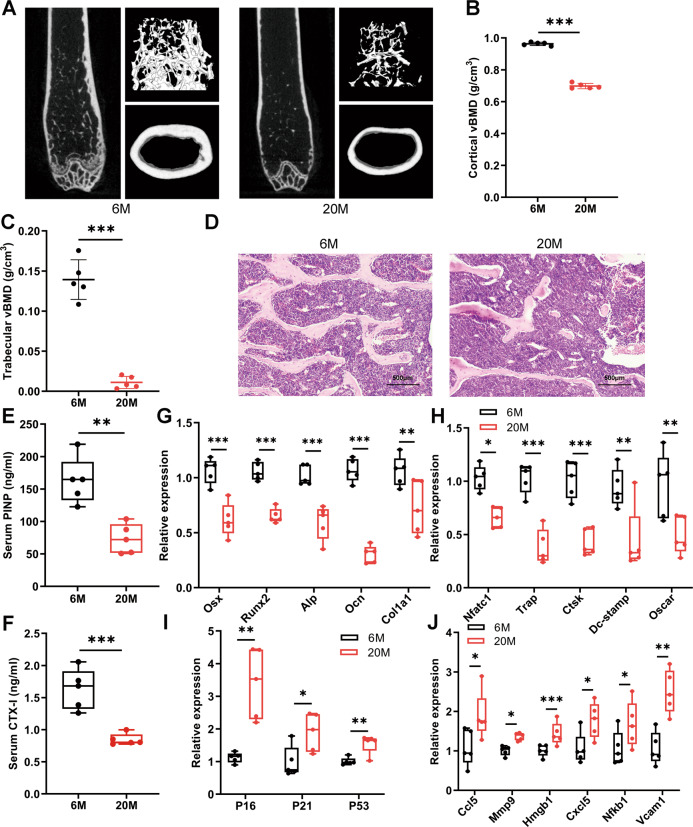
Aged mice exhibit reduced bone mass and aging bone microenvironment. **A** Representative images derived from micro-CT analysis of 6-month-old and 20-month-old mice (*n* = 5). **B**, **C** Quantitative analysis of the volume BMD of cortical bone (**B**) and trabecular bone (**C**) by micro-CT (*n* = 5). **D** Representative images of HE-stained decalcified vertebrae sections of 6-month-old and 20-month-old mice (*n* = 5) (Scale bar, 500 μm). **E**, **F** The levels of serum bone turnover parameters PINP (**E**) and CTX-I (**F**) were detected by ELISA (*n* = 5). **G**, **H** qPCR analysis of osteoblast (**G**) and osteoclast (**H**) differentiation markers in vertebrae (*n* = 5). **I**, **J** qPCR analysis of mRNA levels of senescent genes (**I**) and SASPs (**J**) in vertebrae (*n* = 5). Data presented as mean ± SD. A *t*-test was used for comparison between two groups. vBMD = volumetric bone mineral density. *P < 0.05; **P < 0.01; ***P < 0.001.

Micro-CT analysis revealed serious bone loss of 20-month-old mice when compared with the 6-month-old cohort (Fig. 1A). The volumetric bone mineral densities (vBMDs) of femur significantly decreased in aged mice (Fig. 1B, C). Furthermore, bone microstructure as visualized by hematoxylin–eosin staining (HE staining) of vertebrae slices revealed less and thinner trabecular in old mice (Fig. 1D). To quantify the bone turnover level, procollagen I N-terminal propeptide (PINP) and C-terminal cross-linked telopeptide of type I collagen (CTX-I) in serum, which represent bone formation and resorption [22], respectively, were measured. The results showed that PINP and CTX-I in old mice were both lower than the young, indicating a state of decreased bone remodeling (Fig. 1E, F). We further investigated the expression of key osteoblast and osteoclast differentiation markers in vertebrae and found that they were all decreased in 20-month-old mice, which were consistent with the results of serum biomarkers (Fig. 1G, H).

To verify the aging bone microenvironment, we measured the expression of P16, P21, and P53, which were senescence-associated genes [8, 23], and found that they were elevated in aged mice (Fig. 1I). In addition, a variety of SASPs (Ccl5, Mmp9, Hmgb1, Cxcl5, Nfkb1, and Vcam1) were all highly expressed in aged bone microenvironment(Fig. 1J). These findings clearly indicate that the skeleton exhibits bone loss and senescent features with age.

### BMP9 reduces age-related bone loss and improves bone microenvironment

**Fig. 2:**
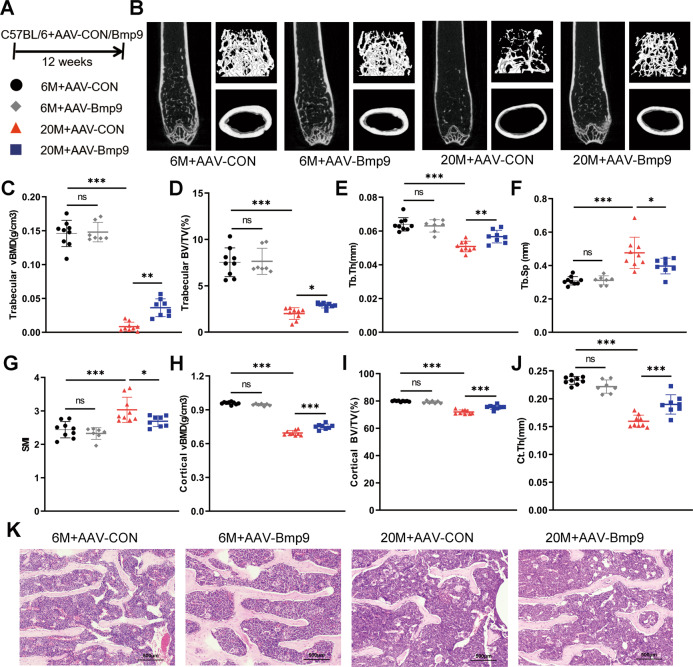
BMP9 reduces age-related bone loss and improves bone microarchitecture. **A** Six-month-old and twenty-month-old mice were injected with AAV-BMP9 or AAV-CON, respectively. Twelve weeks after injection, mice were euthanized for subsequent experiments. **B** Representative images derived from micro-CT analysis, including 2D image construction of distal femur, 3D image reconstruction of trabecular bone of distal femur, and 3D image reconstruction of the femoral midshaft corticoid bone. **C** Quantitative analysis of the vBMD of trabecular by micro-CT. **D**–**G** Microarchitecture analysis of trabecular bone by micro-CT: Trabecular BV/TV (**D**), Tb.Th (**E**), Tb.Sp (**F**) and SMI (**G**). **H** Quantitative analysis of the vBMD of cortical by micro-CT. **I**, **J** Quantitative analysis of the BV/TV (**I**) and Ct.Th (**J**) of corticoid bone by micro-CT. **K** Representative images of HE-stained decalcified vertebrae sections (*n* = 6) (Scale bar, 500 μm). For micro-CT analysis: *n* = 9 for 6 M + AAV-CON group; *n* = 7 for 6 M + AAV-BMP9 group; *n* = 9 for 20 M + AAV-CON group; *n* = 8 for 20 M + AAV-BMP9 group. Data presented as mean ± SD. One-way ANOVA was used for comparison among multiple groups. AAV-CON = empty adeno-associated virus; AAV-BMP9 = BMP9 overexpressing adeno-associated virus; BV/TV = percentage of bone volume; Tb.Th = trabecular thickness, Tb.Sp = trabecular separation, SMI = structure model index, Ct.Th = cortical thickness. *P < 0.05; **P < 0.01; ***P < 0.001. Ns, no significance.

To investigate the impact of BMP9 on bone loss in aged mice, BMP9 overexpressing adeno-associated virus (AAV-BMP9) was injected through tail vein (Fig. 2A). The elevated levels of BMP9 in serum and liver were confirmed 12 weeks later (Fig. S1). Micro-CT analysis revealed that BMP9 overexpression improved the bone mass of old mice, while no effect was observed in the young (Fig. 2B). In aged mice, trabecular vBMD and cortical vBMD were obviously increased after AAV-BMP9 treatment (Fig. 2C, H). Microarchitecture analysis of the trabecular bone in distal femur showed that the percentage of bone (Trabecular BV/TV) and trabecular thickness (Tb.Th) increased in 20-month-old mice with BMP9 overexpression, while trabecular separation (Tb.Sp) and structure model index (SMI) decreased (Fig. 2D–G). BMP9 also increased cortical bone volume (Cortical BV/TV) and cortical thickness (Ct.Th) in aged mice (Fig. 2I, J). HE staining visually showed the increase of trabecular bone in aged mice overexpressing BMP9. However, no difference was observed in the 6-month-old groups (Fig. 2K). These results indicate that BMP9 only reduces bone loss in aged mice, but dose not increase bone mass of the young.

**Fig. 3:**
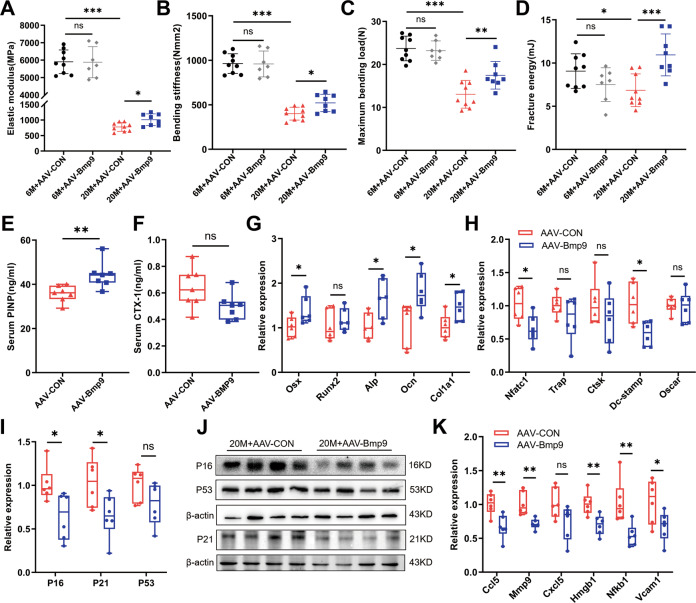
BMP9 improves bone biomechanical performance and bone microenvironment. **A**–**D** The right femur was isolated and subjected to biomechanical properties analysis. The elastic modulus (**A**), bending stiffness (**B**), maximum bending load (**C**), and fracture energy (**D**) were evaluated for each group. **E**, **F** The levels of serum bone turnover parameters PINP (**E**) and CTX-I (**F**) were detected by ELISA (*n* = 7). **G**, **H** qPCR analysis of osteoblast (**G**) and osteoclast (**H**) differentiation markers in vertebrae. (*n* = 6). **I** qPCR analysis of senescent genes in vertebrae (*n* = 6). **J** Western blot analysis of protein levels of senescent genes in vertebrae (*n* = 4). **K** qPCR analysis of SASPs in vertebrae (*n* = 6). For biomechanical properties analysis: *n* = 9 for 6 M + AAV-CON group; *n* = 7 for 6 M + AAV-BMP9 group; *n* = 9 for 20 M + AAV-CON group; *n* = 8 for 20 M + AAV-BMP9 group. Data presented as mean ± SD. A *t*-test was used for comparison between two groups. One-way ANOVA was used for comparison among multiple groups. *P < 0.05; **P < 0.01; ***P < 0.001. Ns, no significance.

Three-point bending test was performed to examine the mechanical properties of the femur. An overall deterioration of the biomechanical properties was observed in aged mice, characterized by markedly reduced elastic modulus, bending stiffness, maximum bending load, and fracture energy. The reduction in mechanical parameters could be improved by BMP9 overexpression in aged mice. Similarly, BMP9 showed no effect on biomechanical parameters in 6-month-old mice (Fig. 3A–D).

To further verify the protective effect of BMP9 in old mice, bone biochemical markers and osteoblast/osteoclast activity were analyzed. Overexpressing BMP9 in aged mice led to elevated serum PINP level and increased osteogenic genes in bone, including Osx, Alp, Ocn, and Col1a1 (Fig. 3E, G). However, there was only a slight decrease in serum CTX-I level, which did not reach significant difference (Fig. 3F). Among the expression of osteoclast differentiation markers, only Nfatc1 and Dc-stamp were downregulated by BMP9 (Fig. 3H). These results indicate that the bone-promoting effect of BMP9 is mainly through facilitating osteogenesis.

It was noteworthy that BMP9 only worked on aged mice, not the young (Fig. S2). It was known that the bone exhibits pro-inflammatory and senescent microenvironment with age [24]. Whether the beneficial effects of BMP9 on bone quality were achieved by inhibiting senescence in bone? To address this question, we detected the expression of key senescent genes in bone microenvironment and found that BMP9 significantly decreased P16 and P21 expression, while no effect on P53 was observed (Fig. 3I, J). Additionally, most of the SASPs (such as Ccl5, Mmp9, Hmgb1, Nfkb1, and Vcam1) expressed in bone microenvironment were also downregulated by BMP9 in aged mice (Fig. 3K). However, the anti-aging effect of BMP9 in young mice was not as obvious as the old (Fig. S3). Taken together, overexpression of BMP9 in vivo improves bone quality and microenvironment only in aged mice and this effect may be related to inhibiting cellular senescence.

### BMP9 attenuates senescence and promotes osteoblast differentiation in vitro

**Fig. 4:**
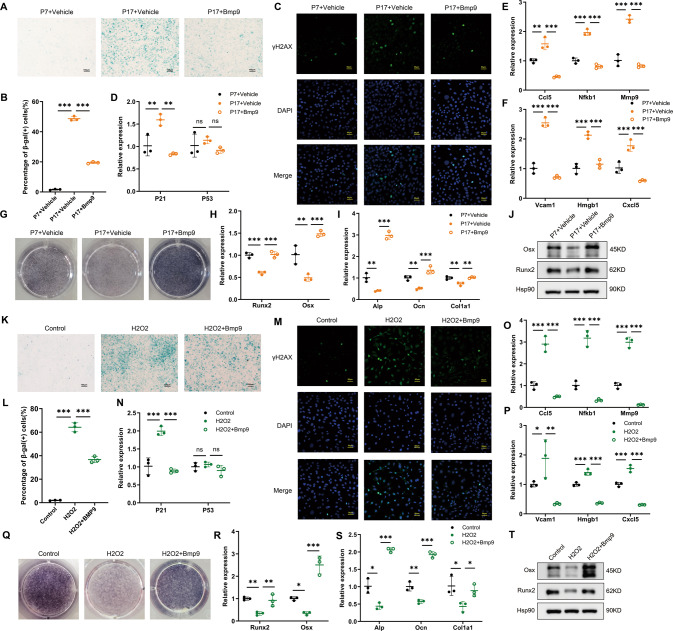
BMP9 attenuates senescence and promotes osteoblast differentiation in MC3T3-E1 cells. Passage 7 of MC3T3-E1 cells were treated with vehicle as control group. Passage 17 of MC3T3-E1 cells were treated with vehicle or BMP9 for 3 days, respectively. **A**, **B** β-galactosidase staining was performed (**A**) and number of β-gal (+) cells was counted for each group (**B**) (Scale bar, 100 μm). **C** Immunofluorescence analysis of γ-H2AX expression (Scale bar, 50 μm). **D** qPCR analysis of mRNA levels of senescent genes P21 and P53. **E**, **F** qPCR analysis of mRNA levels of SASPs. **G** Alp staining of MC3T3-E1 cells suffered to osteogenic differentiation induction for 7 days with or without BMP9. **H**, **I** qPCR analysis of osteoblast differentiation markers in cells suffered to osteogenic differentiation induction for 7 days with or without BMP9. **J** Western blot analysis of protein levels of Runx2 and Osx in MC3T3-E1 cells suffered to osteogenic differentiation induction for 7 days with or without BMP9. Senescence of MC3T3-E1 cells was also induced by H_2_O_2_ treatment for 2 h, then fresh culture medium added with vehicle or BMP9 was replaced for another 3 days. **K**, **L** β-galactosidase staining was performed (**K**) and number of β-gal (+) cells was counted for each group (**L**). (Scale bar, 100 μm). **M** Immunofluorescence analysis of γ-H2AX expression (Scale bar, 50 μm). **N** qPCR analysis of mRNA levels of senescent genes P21 and P53. **O**, **P** qPCR analysis of mRNA levels of SASPs. **Q** ALP staining of control and H_2_O_2_-stimulating MC3T3-E1 cells suffered to osteogenic differentiation induction for 7 days with or without BMP9. **R**, **S** qPCR analysis of osteoblast differentiation markers in control and H_2_O_2_-stimulating MC3T3-E1 cells suffered to osteogenic differentiation induction for 7 days with or without BMP9. **T** Western blot analysis of protein levels of Runx2 and Osx in control and H_2_O_2_-stimulating MC3T3-E1 cells suffered to osteogenic differentiation induction for 7 days with or without BMP9. Data presented as mean ± SD. *n* = 3 biological replicates. One-way ANOVA was used for comparison among multiple groups. *P < 0.05; **P < 0.01; ***P < 0.001. Ns, no significance.

To further evaluate the effect of BMP9 on cellular senescence, we used serial passaging and hydrogen peroxide solution (H_2_O_2_) treatment, respectively, to induce MC3T3-E1 cells senescence in vitro. Subsequently, recombinant mouse BMP9 protein was applied into the culture medium for 3 days. β-galactosidase staining was performed to examine the degree of senescence and the number of β-gal (+) cells was counted. The proportion of β-gal (+) cells reached 48.69% and 64.16% in the passage 17 of MC3T3-E1 cells and cells treated with H_2_O_2_, respectively. Treatment with BMP9 significantly decreased this proportion to 19.45% in repeating passaged and 36.65% in H_2_O_2_-induced MC3T3-E1 cells (Fig. 4A, B, K, L). In addition, the markers of DNA damage response γ-H2AX were increased in MC3T3-E1 cells after senescence induction, and were significantly downregulated by BMP9 (Fig. 4C, M). Moreover, the expression of P21 was significantly increased in senescent cells, while P53 unchanged (Fig. 4D, N), indicating that senescence of MC3T3-E1 cells might be regulated mainly by P21 rather than P53. BMP9 suppressed the expression of P21, accompanied with decreased SASPs (Fig. 4E, F, O, P). The above results indicate that BMP9 inhibits senescence of MC3T3-E1 cells in different senescence-inducing conditions.

Furthermore, senescent MC3T3-E1 cells showed deteriorated osteogenic potential as evidenced by impaired alkaline phosphatase staining (Alp staining) and decreased expression of osteoblast differentiation markers Runx2, Osx, Alp, Ocn, and Col1a1 after 7 days osteogenic induction. The impaired osteogenic ability of senescent osteoblast could be rescued by BMP9 treatment (Fig. 4G–J, Q–T). These results suggest that BMP9 promotes osteogenic differentiation in senescent osteoblast at least partially through attenuating cellular senescence.

As Alk1 is a specific receptor for BMP9 ligand [25], we next knocked down Alk1 on MC3T3-E1 cells to examine the effect of Alk1-deficiency on osteoblast senescence induced by H_2_O_2_. Alk1 knockdown efficiency was confirmed by quantitative polymerase chain reaction (qPCR) and Western blot (Fig. S4A, B). After H_2_O_2_ treatment, Alk1-deficient cells showed significantly increased expression of P21 (Fig. S4C, D) and SASPs (Fig. S4E). The results of β-galactosidase staining indicated that the proportion of senescent cells in Alk1-deficient osteoblasts was much higher than the control cells (Fig. S4F, G). Moreover, after 7 days of osteoblastic induction, the Alp staining and the expression of key osteoblast differentiation genes suggested that Alk1 knockdown further impaired the differentiation capacity of senescent osteoblasts (Fig. S4H, I). These results demonstrate that blocking the BMP9 signaling pathway through knocking down its receptor Alk1 accelerates osteoblast senescence in vitro. Furthermore, we detected the expression of Alk1 in bone of 6-month and 20-month-old mice, respectively, and found a significantly reduction in aged mice (Fig. S5), which suggested a low active state of BMP9 signaling in vivo.

### Stat1 is a key factor in regulating senescence of osteoblast

According to the above results, we demonstrated that BMP9 had the potential of suppressing senescence and promoting osteogenesis. To further explore the specific molecular mechanism, RNA sequencing analysis of senescent MC3T3-E1 cells treated with or without BMP9 was applied to uncover the differentially expressed genes.

**Fig. 5:**
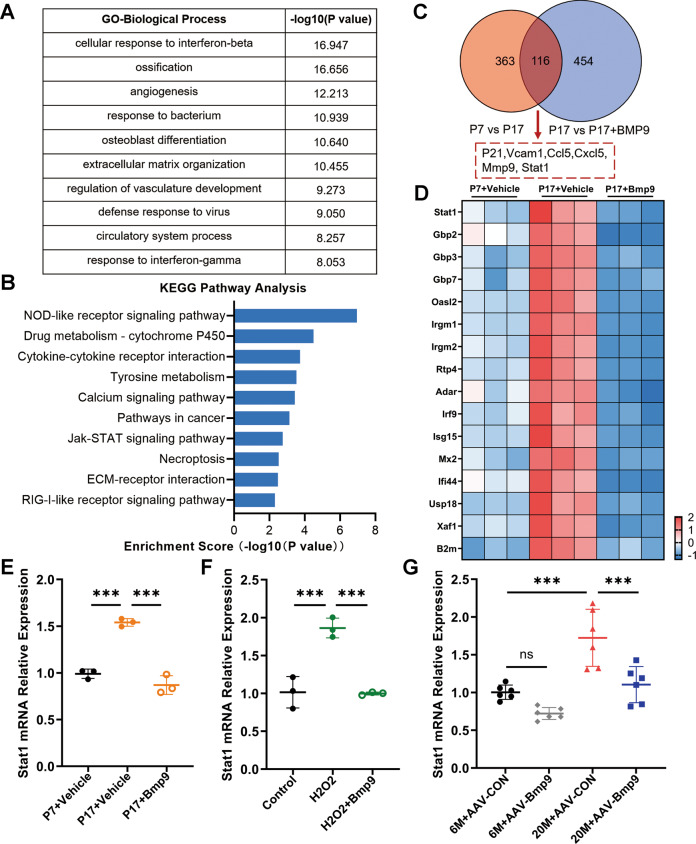
Stat1 is a key factor in regulating senescence of osteoblast. **A** GO analysis of differentially expressed genes between replicative senescent MC3T3-E1 cells treated with vehicle or BMP9 for 3 days. **B** Top 10 downregulated pathways in KEGG analysis. **C** Overlap analysis of upregulated genes between normal control with replicative senescent MC3T3-E1 cells and downregulated genes between senescent MC3T3-E1 cells treated with vehicle or BMP9. **D** Heatmap of Stat1 and its downstream target genes in passage 7 and passage 17 of MC3T3-E1 treated with vehicle or BMP9 for 3 days. **E** qPCR analysis of mRNA level of Stat1 in cells of passage 7 and passage 17 with vehicle or BMP9 treatment for 3 days. **F** qPCR analysis of mRNA level of Stat1 in control and H_2_O_2_-stimulating MC3T3-E1 cells treated with vehicle or BMP9 for 3 days. **G** qPCR analysis of mRNA levels of Stat1 in 6-month-old and 20-month-old mice treated with AAV-CON or AAV-BMP9 (*n* = 6). Data presented as mean ± SD. *n* = 3 biological replicates for in vitro experiment. One-way ANOVA was used for comparison among multiple groups. *P < 0.05; **P < 0.01; ***P < 0.001. Ns, no significance.

Gene Ontology analysis revealed that genes regulated by BMP9 were associated with essential biological processes of osteoblast, such as ossification, osteoblast differentiation, and extracellular matrix organization (Fig. 5A). Among the top 10 biological processes, cellular responses to interferon-beta and interferon-gamma were associated with various SASPs production and senescence [26], and were downregulated by BMP9 (Table S1). KEGG pathway analysis further displayed that the typical interferon-related signaling pathway Jak-Stat which included a series of pro-inflammatory factors was one of the most profound pathways downregulated by BMP9 (Fig. 5B). Among these downregulated genes, Stat1 is a crucial transcription factor regulating the expression of P21 and multiple SASPs [27–29]. We further analyzed the overlap of 479 highly expressed genes in senescent MC3T3-E1 cells and 570 genes that were downregulated by BMP9. Totally 116 genes were in this category, including Stat1 and its downstream target genes (Fig. 5C, D).

The expression of Stat1 was further examined in senescent MC3T3-E1 cells with or without BMP9 treatment. Both serial passaging and H_2_O_2_-stimuli elevated the expression of Stat1, which could be abolished by BMP9 (Fig. 5E, F). There was also a remarkable increase of Stat1 expression in bone samples from aged mice, which was downregulated by AAV-BMP9 treatment (Fig. 5G). These findings suggest that BMP9 can suppress the expression of Stat1.

### The suppression of Stat1 is crucial for the anti-senescence effect of BMP9 in osteoblast

**Fig. 6:**
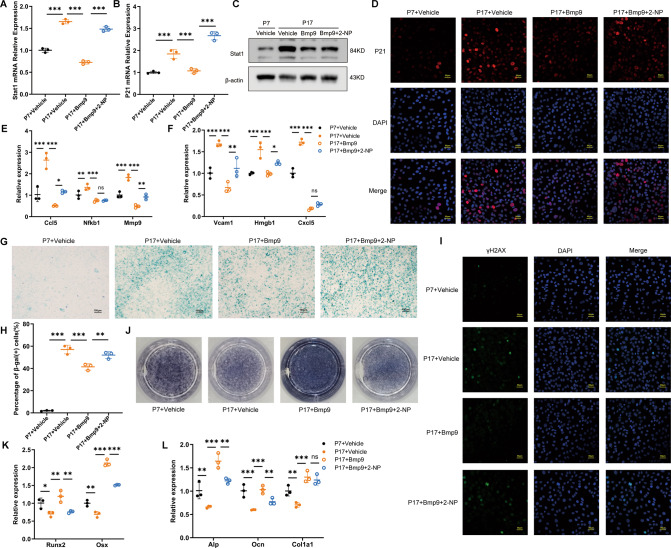
The suppression of Stat1 is crucial for the anti-aging effect of BMP9 in replicative senescence of osteoblast. Passage 7 of MC3T3-E1 cells were set as control group, passage 17 of MC3T3-E1 cells were treated with vehicle or BMP9 or BMP9 + 2-NP for 3 days, respectively. **A**, **B** qPCR analysis of Stat1 and P21 expression. **C** Western blot analysis of protein level of Stat1. **D** Immunofluorescence analysis of protein level of P21. (Scale bar, 50 μm). **E**, **F** qPCR analysis of mRNA levels of SASPs. **G**, **H** β-galactosidase staining was performed (**G**) and number of β-gal (+) cells was counted for each group (**H**) (Scale bar, 100 μm). **I** Immunofluorescence analysis of γ-H2AX expression (Scale bar, 50 μm). **J** Alp staining of MC3T3-E1 cells suffered to osteogenic differentiation induction for 7 days. **K**, **L** qPCR analysis of mRNA levels of osteoblastic differentiation markers. Data presented as mean ± SD. *n* = 3 biological replicates. One-way ANOVA was used for comparison among multiple groups. *P < 0.05; **P < 0.01; ***P < 0.001. Ns, no significance.

**Fig. 7:**
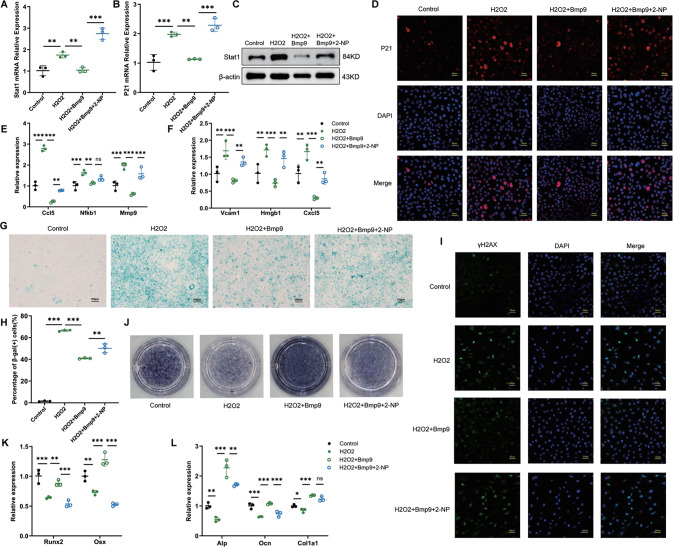
The suppression of Stat1 is crucial for the anti-aging effect of BMP9 in H_2_O_2_-induced senescence of osteoblast. Senescence of MC3T3-E1 cells was induced by H_2_O_2_ treatment for 2 h, then fresh culture medium added with vehicle or BMP9 or BMP9 + 2-NP was replaced for another 3 days. **A**, **B** qPCR analysis of Stat1 and P21 expression. **C** Western blot analysis of protein level of Stat1. **D** Immunofluorescence analysis of protein level of P21. (Scale bar, 50 μm). **E**, **F** qPCR analysis of mRNA levels of SASPs. **G**, **H** β-galactosidase staining was performed (**G**) and number of β-gal (+) cells was counted for each group (**H**) (Scale bar, 100 μm). **I** Immunofluorescence analysis of γ-H2AX expression (Scale bar, 50 μm). **J** Alp staining of MC3T3-E1 cells suffered to osteogenic differentiation induction for 7 days. **K**, **L** qPCR analysis of mRNA levels of osteoblastic differentiation markers. Data presented as mean ± SD. *n* = 3 biological replicates. One-way ANOVA was used for comparison among multiple groups. *P < 0.05; **P < 0.01; ***P < 0.001. Ns, no significance.

To further verify the importance of Stat1 suppression in mediating the anti-senescence effect of BMP9, a rescue experiment was performed by applying a specific transcriptional activator of Stat1 termed as 2-(1,8-Naphthyridin-2-ly)phenol (2-NP) [30]. Activation of Stat1 was confirmed by elevated mRNA and protein expression, accompanied with increased P21 after 2-NP application (Fig. 6A–D, Fig. 7A–D). The SASPs (Ccl5, Mmp9, Vcam1, and Hmgb1) reduced by BMP9 were also increased with 2-NP treatment (Figs. 6E, F, 7E, F). The proportion of β-gal (+) cells increased from 41.4% to 52.01% in serial passaged cells with 2-NP (Fig. 6G, H). In H_2_O_2_-treated cells, the percentage was reduced to 40.9% by BMP9 and increased back to 50.14% under 2-NP treatment (Fig. 7G, H). Consistently, nuclear γ-H2AX expression was also elevated by 2-NP in both senescent groups in the presence of BMP9 (Figs. 6I, 7I).

The osteogenic potential of senescent MC3T3-E1 cells treated with BMP9 and 2-NP was examined. Alp staining of senescent osteoblast in BMP9 + 2-NP group was weaker than the BMP9 group (Figs. 6J, 7J). The elevated expression of differentiation markers in BMP9-treated cells was also reduced by 2-NP (Figs. 6K, L, 7K, L). These results indicate that the anti-senescence effect of BMP9 in osteoblast depends on its inhibition on Stat1.

### BMP9 inhibits osteoblast senescence through Smad1-Stat1-P21 axis

**Fig. 8:**
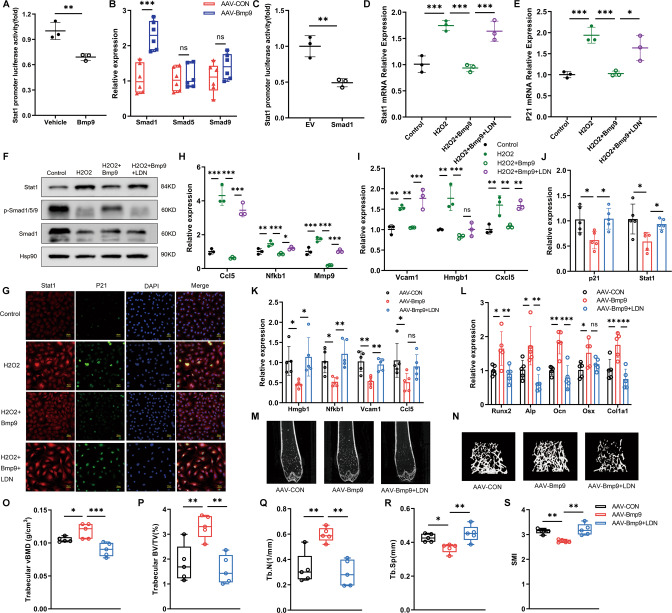
BMP9 inhibits osteoblast senescence through Smad1-Stat1-P21 axis. **A** Relative Stat1 promotor luciferase activity after treated with vehicle or BMP9 for 24 h (*n* = 3 biological replicates). **B** qPCR analysis of mRNA levels of Smad1, Smad5, and Smad9 in vertebrae of 20-month-old mice treated with AAV-CON or AAV-BMP9 (*n* = 6). **C** MC3T3-E1 cells were co-transfected with Stat1 promoter reporter plasmid and empty vector or Smad1-expressing vector for 36 h. The relative Stat1 promotor luciferase was examined (*n* = 3 biological replicates). **D**, **E** qPCR analysis of mRNA levels of Stat1 (**D**) and P21 (**E**) of control and H_2_O_2_-induced MC3T3-E1 cells treated with BMP9 or BMP9 + LDN193189 for 3 days (*n* = 3 biological replicates). **F** Western blot analysis of protein levels of Stat1, p-Smad1/5/9, and total Smad1 in control and H_2_O_2_-induced MC3T3-E1 cells treated with BMP9 or BMP9 + LDN193189 (*n* = 3 biological replicates). **G** Immunofluorescence analysis of P21(+) Stat1 (+) cells (Scale bar, 50 μm). **H**, **I** qPCR analysis of mRNA levels of SASPs in control and H_2_O_2_-induced MC3T3-E1 cells treated with BMP9 or BMP9 + LDN193189 (*n* = 3 biological replicates). **J** qPCR analysis of mRNA levels of P21 and Stat1 in vertebrae (*n* = 5). **K** qPCR analysis of mRNA levels of SASPs in vertebrae (*n* = 5). **L** qPCR analysis of mRNA levels of osteoblastic markers in vertebrae (*n* = 5). **M**, **N** Representative images derived from micro-CT analysis, including 2D image construction of distal femur (**M**) and 3D image reconstruction of trabecular bone of distal femur (**N**). **O**–**S** Quantitative analysis of the vBMD (**O**) and microarchitecture analysis of trabecular bone by micro-CT: Trabecular BV/TV (**P**), Tb.N (**Q**), Tb.Sp (**R**) and SMI (**S**) (*n* = 5). Data presented as mean ± SD. One-way ANOVA was used for comparison among multiple groups. *P < 0.05; **P < 0.01; ***P < 0.001. Ns, no significance.

According to the above findings, BMP9 attenuates cellular senescence by downregulation of Stat1, leading to reduced P21 expression. We then explored the regulatory mechanism of BMP9 on Stat1. Dual-luciferase reporter assay showed that treatment with BMP9 for 24 h significantly decreased the activity of Stat1 promotor (Fig. 8A). Since Smad1/5/9 is widely involved in the regulatory effect of BMP9 [31, 32], we detected their expression in bone of old mice, finding that the expression of Smad1 was significantly elevated by BMP9 overexpression (Fig. 8B). We then co-transfected Stat1 promoter reporter plasmid and Smad1-expressing vector in MC3T3-E1 cells for 36 h. The luciferase activity of Stat1 was obviously downregulated, indicating the inhibitory effect of Smad1 on Stat1 promoter activity (Fig. 8C).

With the use of Smad1/5/9 signaling pathway inhibitor LDN193189 [33], activation of Smad1 induced by BMP9 was inhibited, resulting in increased expression of Stat1 and P21 in H_2_O_2_-induced senescent cells (Fig. 8D–F). The number of P21 (+) Stat1 (+) cells was also increased with LDN193189 (Fig. 8G). Moreover, reduction of Ccl5, Nfkb1, Mmp9, Vcam1 and Cxcl5 in BMP9-treated senescent osteoblasts was partially neutralized by the application of LDN193189 (Fig. 8H, I).

To further investigate the effect of Smad1 inhibition in vivo, aged mice with BMP9 overexpression were treated with LDN193189 injection. The decreased expression of P21 and Stat1 in the bone of BMP9-overexpressed mice was upregulated by LDN193189 intervention (Fig. 8J). In addition, LDN193189 reversed the inhibitory effect of BMP9 on SASPs in vivo, which was manifested by increased expression of Hmgb1, Nfkb1, and Vcam1 (Fig. 8K). Furthermore, the expression of osteoblast-specific genes in vertebrae of aged mice was upregulated by BMP9 and downregulated by LDN193189 (Fig. 8L). Micro-CT showed that LDN193189 eliminated the improvement on bone mass and trabecular microarchitecture of aged mice caused by BMP9 overexpression (Fig. 8M–S). These results suggest that BMP9 inhibits cellular senescence by activation of Smad1, which suppresses the promoter activity of Stat1, resulting in decreased P21 expression and SASPs production in osteoblast. Interfering with the Smad1 signal can reverse the anti-aging effect of BMP9 and undermine the protection of BMP9 on age-related bone loss.

## Discussion

In current study, we provide novel evidence that BMP9 reduces bone loss and improves senescent bone microenvironment in aged mice. We further reveal that BMP9 inhibits expression of senescent gene and SASPs in osteoblast through Smad1-Stat1-P21 axis, leading to improved osteoblastogenic differentiation. Our work uncovers a new mechanism for the protection effect of BMP9 on age-related bone loss.

Age-related osteoporosis is characterized by significantly decreased number of osteoblasts, which has been partially attributed to senescence of osteoblastic lineage cells [9, 34]. Eliminating or reducing the burden of senescent cells represents a valuable therapeutic approach to age-related osteoporosis [3]. Based on this conception, our study aims to verify the anti-aging effect of BMP9 and further explore the molecular mechanisms. In this study, we find two features of BMP9’s regulation on age-related bone loss. First, the protective effect of BMP9 is observed only in aged mice, evidenced by elevated bone quality and decreased expression of senescent genes and SASPs after BMP9 overexpression in vivo. However, these beneficial effects are not observed in young mice receiving similar treatment. Consistently, recent study noted that mice treated with senolytic drugs (Dasatinib + Quercetin) from 18-month-old to 23-month-old showed improved vertebral bone quality parameters, whereas same treatment during 6–23 months of age showed no obvious effect [14]. It is suggested that an optimal treatment window is necessary for BMP9 when senescent cells accumulating within bone microenvironment and affecting bone homeostasis during aging. Second, the improvement on bone mass accomplished by BMP9 is mainly through regulating bone formation by suppressing osteoblast senescence rather than inhibiting bone resorption, which is inconsistent with the dual regulation of BMP9 in OVX-induced osteoporosis [35]. Since the cardinal feature of age-related osteoporosis is the accumulation of senescent osteoblastic lineage cells in bone microenvironment, rather than a profound increase in the activity of osteoclast, eliminating senescent osteoclast progenitors cannot alleviate age-associated bone loss [36]. Therefore, targeting senescent osteoblastic cells is effective for treatment of osteoporosis caused by aging.

In this study, BMP9 inhibited senescence of MC3T3-E1 cells after multiple passages, which was evidenced by reduced number of β-gal (+) cells and decreased expression of senescence markers. For serial passaging causing replicative senescence [37], it was used to study senescence of primary cells in previous studies [38, 39]. To reconfirm our findings, another senescent cell model was established using H_2_O_2_, which was widely used to induce senescence in different cell lines [40, 41]. BMP9 exhibited similar suppression of senescent phenotype in both senescence-inducing conditions, followed by promoted osteogenic differentiation. In addition, we also found that osteoblasts lacking the BMP9-specific receptor Alk1 were more prone to senescence induced by H_2_O_2_ with a further degenerated differentiation capacity. Furthermore, we also detected the serum BMP9 level and the expression of Alk1 in vertebrae from both young and old mice. Consistently, the content of BMP9 in serum and the expression of Alk1 in bone of aged mice were significantly decreased, which represented a low active state of BMP9 signal in vivo. Combined with the results of reduced bone mass in aged mice and the improvement after BMP9 overexpression, we cautiously make the point that BMP9 does have an inhibitory effect on senescent osteoblasts, which contributes to its osteogenic promoting effect.

After revealing the anti-senescence effect of BMP9, we further explored the potential mechanism using RNA sequencing analysis [42, 43]. The result showed markedly increased expression of Stat1 and its target genes in senescent osteoblast, which was consistent with prior studies showing the pro-aging effect of Stat1 [27, 28, 44]. It has been noted that Stat1 can induce cell cycle arrest by enhancing the transcriptional activity of P21 as a transcription factor [44, 45]. Our work showed that the expression of Stat1 was significantly downregulated by BMP9 in senescent osteoblast in vitro and aged bone microenvironment in vivo, indicating the novel role of BMP9 on regulating Stat1. To further evaluate the role of Stat1, we used a specific activator of Stat1 in the presence of BMP9 on senescent osteoblast, and found that the senescent phenotype was recovered along with deteriorated osteogenic differentiation when comparing with cells treated with BMP9 alone. Therefore, we provide evidence that BMP9 inhibits senescence by downregulating the expression of Stat1, resulting in decreased P21 expression.

Previous studies have shown that Smad1 is a key effector downstream of BMP9, and is widely involved in various essential biological processes [19, 31]. However, the effects of Smad1 on senescence and age-related osteoporosis are rarely investigated. It was reported that Smad1 deletion led to increased P16 expression and shortened lifespan of osteoblast [46]. Consistently, we showed the reduction of P16 by BMP9 overexpression in vertebrae of aged mice. In addition, we found that another essential senescent gene P21 was also downregulated by BMP9 accompanied with elevated Smad1 expression. As P21 is a target gene of Stat1 [26, 47, 48], our work provides further evidence that there does exist an novel axis between Smad1 and Stat1/P21 signal in osteoblast. Previous studies clarified the roles of Smad1 and Stat1 in proliferation, differentiation, and functional regulation of different cells [27, 29, 32]; however, the interactions between them need to be further investigated. This study uncovered a novel role for Smad1 in repressing the transcriptional activity of Stat1, leading to decreased P21 and SASPs production in osteoblasts. Moreover, we performed in vivo experiments to demonstrate that interfering with the Smad1 signal reversed the inhibitory effect of BMP9 on Stat1 and downstream senescent genes, which undermined the protection of BMP9 on age-related bone loss. Thus, in this study, from in vitro to in vivo, at cellular and molecular levels, we provided series evidence from multiple aspects to demonstrate the Smad1-Stat1-P21 signaling pathway regulated by BMP9, which was helpful to expand the network of interactions between Smad1 and Stat1. Given the complexity and diversity of molecular interactions in different cells, more in-depth mechanisms about mutual regulation between Smad family and Stat1 need further investigation.

In summary, our work highlights the critical role of BMP9 in reducing age-related bone loss and improving bone quality by inhibiting senescence in bone microenvironment. The anti-aging effect of BMP9 is achieved by downstream Smad1, which suppresses transcriptional activity of Stat1, leading to decreased expression of P21 and SASPs in senescent osteoblast. Our finding not only provides theoretical support for anti-aging treatment on osteoporosis but also enriches the therapeutic value of BMP9 as translational medicine in the future.

## Materials and methods

### Animals

Male C57BL/6 mice of 6 months (*n* = 16) and 20 months (*n* = 17) were arranged as young and old, respectively. The BMP9 overexpression model was established as previously described [49, 50]. Briefly, 6-month and 20-month-old mice were both received a tail vein injection of empty adeno-associated virus (AAV-CON) or AAV-BMP9, 5 × 10^11^ vg/mouse, once. For the aged mice received both AAV-BMP9 and LDN193189 treatment, 3 mg/kg LDN193189 was used once every other day by intraperitoneal injection. The same volume of normal saline was given to control groups. Animals were randomly assigned to experimental groups. Twelve weeks after AAV injection, mice were euthanized. Elevated level of BMP9 expression in liver and serum was confirmed. Lumbar spines and femurs were collected for subsequent experiments. Mice were housed at 22 ± 2 °C and 55–60% humidity a specific pathogen-free environment, given water and a standard rodent chow diet with a 12-h light/dark cycle. All animal experiments were conducted according to the Guide for the Care and Use of Laboratory Animals (National Institutes of Health publication 85–23, revised 1996) and were approved by Shanghai Jiao Tong University School of Medicine Animal Study Committee.

### Micro-CT analysis and three-point bending test

All procedures involving micro-CT were performed according to the recommendation of the American Society for Bone and Mineral Research [51]. Briefly, the right femurs of mice were isolated, fixed in 4% paraformaldehyde for 48 h, and then maintained in 75% ethanol. Quantitative analysis of distal femoral metaphysis and midshaft femoral diaphysis were performed using a high-resolution ex vivo micro-CT scanner (SkyScan 1176, Bruker, Kontich, Belgium). Using 2D data from scanned slices, 3D analysis was performed to calculate morphometric parameters by a CT scan software (Ctan, Bruker, Kontich, Belgium). The following trabecular morphometric indexes were analyzed: volumetric bone mineral density (vBMD), percentage of bone volume (BV/TV), trabecular number (Tb.N), trabecular thickness (Tb.Th), trabecular separation (Tb.Sp) and structure model index (SMI). The cortical thickness (Ct.Th) was assessed at the midshaft of femurs. All the analyses were conducted in a blinded fashion.

For biomechanical testing, the left femurs were cleaned of adhered tissue, wrapped by saline-soaked gauze, and tested immediately. Three-point bending test was carried out at the midshaft of the femurs on a mechanical-testing machine (Instron 5569; Instron, Inc., Grove City, PA, USA). The elastic modulus, bending stiffness, maximum bending load, and fracture energy were evaluated. All the analyses were conducted in a blinded fashion.

### Measurement of serum biomarkers

Mice were fasted for at least 12 h before euthanized. Blood was collected and allowed to clot at room temperature for 1 h, then centrifuged at 4000 rpm for 15 min at 4 °C. Serum samples were stored at −80 °C. Enzyme linked immunosorbent assay (ELISA) was employed to determine levels of serum PINP, CTX-I (USCN Life Science, Wuhan, China), and BMP9 (RayBiotech, Peachtree Corners, GA, USA) according to the manufacturer’s introductions. All the analyses were conducted in a blinded fashion.

### HE staining

The fixed lumbar spines of mice were decalcified in 23% ethylenediaminetetraacetic acid at 4 °C for 5–7 days and embedded in paraffin. Bone sections (4 μm) were deparaffinized, and washed with water for 3 min. Then hematoxylin solution (Beyotime, Shanghai, China) was applied for 10 min, washed with water, hydrochloric acid ethanol for 30 s, counterstained with eosin solution, and dehydrated. All the analyses were conducted in a blinded fashion.

### Cell culture, osteogenic induction, and reagent treatment

Mycoplasma-free osteoblastic MC3T3-E1 cells (American Type Culture Collection, Manassas, VA, USA), a widely used clonal osteoblast-like cell line isolated from calvaria of embryonic mouse [52, 53], was cultured in α-modified minimal essential medium (Gibco, Thermo Fisher Scientific, Waltham, MA, USA) supplemented with 10% fetal bovine serum and 1% penicillin/streptomycin at 37 °C with 5% CO2. The culture medium was changed every 2 days. For osteogenic induction, 10 mM β-glycerophosphate disodium salt hydrate (Sigma-Aldrich, St. Louis, MO, USA) and 50 μM ascorbic acid (Sigma-Aldrich, St. Louis, MO, USA) was added into the growth medium. Cells of passage 7 were classified as the normal control group, while passage 17 as the replicative senescent group. 200 μM H_2_O_2_ and 100 ng/ml BMP9 (R&D Systems, Minneapolis, MN, USA) were used to induce or inhibit cellular senescence. The same volume of phosphate buffer saline was given to control groups. To block the downstream pathways of BMP9, 50 μM 2-NP (Abcam, Cambridge, UK) and 0.5 μM LDN193189 (Selleck Chemicals, Houston, TX, USA) were added to the cell culture medium at the same time with BMP9, respectively. Information of cell line used in this study is shown in Table S2. Information of biological modulators is listed in Table S3.

### β-galactosidase staining assay

Cells were stained following the manual of the Senescence β-galactosidase Staining Kit (Beyotime, Shanghai, China). In brief, cells with different treatments were harvested and washed, then fixed and stained with the β-galactosidase staining solution at 37 °C overnight. Level of senescence was quantified by visual examination of blue-stained cells and the number of β-gal (+) cells were counted with an inverted microscope. The experiment was replicated three times.

### Western blot and immunofluorescence assay

Total protein lysates were obtained using RIPA buffer (Biocolors, Shanghai, China) supplemented with Protease Inhibitor Cocktail (APExBIO, Suzhou, China). After denaturation, the protein lysates were subjected to SDS-PAGE and transferred onto a polyvinylidene difluoride membrane (Millipore, Burlington, MA, USA). Afterward, membranes were blocked in 5% fat-free milk (Sangon Biotech, Shanghai, China) for 1 h at room temperature and then incubated with the primary antibodies against BMP9, Osx, P16, Alk1 (1:1000 dilution, Abcam, UK), P21, P53, Runx2, Stat1, Smad1, p-Smad1/5/9, β-actin (1:1000 dilution, CST, USA) and HSp90 (1:1000 dilution, Santa Cruz Biotechnology, USA) overnight at 4 °C. HRP-conjugated secondary antibodies were incubated for 1 h at room temperature. The protein bands were detected with enhanced chemiluminescence reagents using the eBlot Touch Imager (eBlot, Shanghai, China). The experiment was replicated three times.

For immunofluorescence assay, cells were cultured on coverslips, washed with phosphate buffer saline, and fixed with 4% paraformaldehyde for 15 min. After permeabilized in 0.5% Triton X-100 and blocked with 20% goat serum, the slides were incubated with primary antibodies against γ-H2AX (1:400 dilution, CST, USA), Stat1 (1:400 dilution, CST, USA) and P21 (1:100 dilution, Santa Cruz Biotechnology, USA) overnight at 4 °C. Afterward, the cells were incubated with Alexa Fluor 488-conjugated or Alexa Fluor 594-conjugated secondary antibodies (1:500 dilution, CST, USA) for 1 h at room temperature and mounted with DAPI Fluoromount-G mounting media (SouthernBiotech, Birmingham, AL, USA). Images were acquired using a Zeiss LSM880 confocal microscope (Zeiss, Inc., Thornwood, NY, USA). The experiment was replicated three times. The information of primary antibodies was shown in Table S4.

### Total RNA extraction and qPCR analysis

Total RNA was extracted from tissues or cells using the RNA isolator Total RNA Extraction Reagent (Vazyme, Shanghai, China). The RNA concentration and absorbance ratio at 260/280 nm of all samples were detected using a NanoDrop ND2000 spectrophotometer (Thermo Scientific, Waltham, MA, USA). Reverse transcription was performed using the PrimeScript™ Reverse TranscriptMasterMix (TaKaRa Bio, Otsu, Japan). qPCR was conducted using the QuantStudio™Dx Real-Time PCR Instrument (Applied Biosystems, Foster City, CA, USA). The ΔΔCT method was used to evaluate the relative mRNA expression which was normalized to β-actin. The experiment was replicated three times. Primer sequences are listed in Table S5.

### Alp staining assay

Alp staining of differentiated MC3T3-E1 cells was performed using a 5-bromo-4-chloro-3-indolyl phosphate/tetranitro blue tetrazolium chloride (BCIP/NBT) alkaline phosphatase color development kit (Beyotime, Shanghai, China) according to the manufacturer’s instructions. In brief, MC3T3-E1 cells were washed and fixed with 4% paraformaldehyde following the 7 days differentiation induction, and the staining solution was applied for 5-15 min at room temperature. Level of differentiation was quantified by visual examination of cells stained purple. The experiment was replicated three times.

### Lentivirus infection

Lentivirus-based Alk1 short hairpin RNA (shRNA) was purchased from Shanghai Xitubio Biotechnology Co., Ltd (Shanghai, China). The shRNA targeting sequence was 5'-ACCTACATGTGGAGATCTTTG-3'. Lentivirus was infected at a multiplicity of infection (MOI) of 80 with 6 μg/ml polybrene for 24 h. After 48 h of infection, culture medium containing puromycin (2 ug/ml) was used to acquire stably transfected cells. Alk1 gene knockdown was verified by qPCR and Western blot analysis.

### RNA sequencing analysis

One microgram total RNA from each sample was prepared and the quality of isolated RNA was validated by Agilent 2100 bioanalyzer (Agilent, Santa Clara, California, USA). cDNA libraries were constructed using NEBNext Ultra™ RNA Library Prep Kit (Illumina, San Diego, California, USA) and sequencing was performed on IlluminaHiSeq 4000 (Illumina, San Diego, California, USA). After quality control, raw sequencing data was pretreated into trimmed data and further mapped to mouse genome reference using HISAT2. The differentially expressed genes and transcripts were identified by setting a threshold at fold change ≥ 2.0, *p*-value < 0.05.

### Plasmids transfection and dual-luciferase reporter assay

The mouse Stat1 promoter region (−2000 to +50 from transcriptional start site) was cloned into PGL3-basic-Luc vector. Plasmids encoding Smad1 were constructed using PSC2 + vector. All constructs were verified by DNA sequencing. To explore the effect of BMP9 on Stat1 expression, MC3T3-E1 cells were transfected with pRL-SV40 plasmid (expressing Renilla luciferase) and Stat1 promoter reporter constructs for 24 h, and then recombinant mouse BMP9 protein was added into the culture medium for another 24 h, followed by luciferase activity measurement. Furthermore, MC3T3-E1 cells were co-transfected with pRL-SV40 plasmid, Stat1 promoter reporter construct, and Smad1-expressing vector for 36 h. Luciferase activity was analyzed on a GloMax 20/20 Luminometer (Promega Corporation, Madison, WI, USA). The experiment was replicated three times.

### Statistical analysis

All experimental data are presented as mean ± SD. Statistical significance was tested using a two-tailed Student’s *t*-test between two groups and one-way ANOVA followed by a Fisher’s least significant difference post hoc test was performed when comparing more than two groups. Statistical analysis was performed using SPSS 25.0. *P*-value < 0.05 was considered statistically significant.

## Supplementary information

checklist

supplementary figures

supplementary tables

original WB

## Data Availability

The datasets used during the current study are available from the corresponding author on reasonable request.
