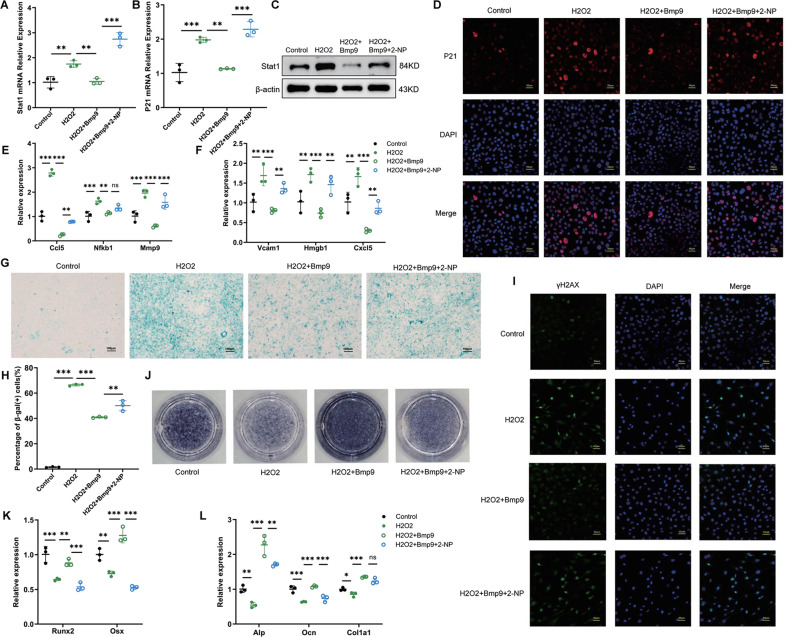# Correction to: BMP9 reduces age-related bone loss in mice by inhibiting osteoblast senescence through Smad1-Stat1-P21 axis

**DOI:** 10.1038/s41420-022-01128-9

**Published:** 2022-07-22

**Authors:** Jing-zun Xu, Yan-man Zhou, Lin-lin Zhang, Xiao-jing Chen, Yu-ying Yang, Deng Zhang, Ke-cheng Zhu, Xiao-ke Kong, Li-hao Sun, Bei Tao, Hong-yan Zhao, Jian-min Liu

**Affiliations:** 1 Department of Endocrine and Metabolic Diseases, Shanghai Institute of Endocrine and Metabolic Diseases, Ruijin Hospital, Shanghai Jiao Tong University School of Medicine, Shanghai, China; 2 Shanghai National Clinical Research Center for Metabolic Diseases, Key Laboratory for Endocrine and Metabolic Diseases of the National Health Commission of the PR China, Shanghai Key Laboratory for Endocrine Tumor, State Key Laboratory of Medical Genomics, Ruijin Hospital, Shanghai Jiao Tong University School of Medicine, Shanghai, China; 3 Department of Nephrology, Provincial Hospital affiliated to Shandong First Medical University, Jinan, Shan Dong Province China

**Keywords:** Osteoporosis, Ageing

Correction to: *Cell Death Discovery* 10.1038/s41420-022-01048-8, published online 06 May 2022

The original version of this article unfortunately contained an error in Figure 7. The authors apologize for the mistake. The wrong immunofluorescence image was used in the position of H2O2+BMP9 group in Figure 7I. The correct figure can be found below. The original article has been corrected.