# Supplementary material for: BMP9 reduces age-related bone loss in mice by inhibiting osteoblast senescence through Smad1-Stat1-P21 axis
Source: Cell Death Discov. 2022 May 6;8:254. doi: 10.1038/s41420-022-01048-8 (PMC9076651; doi:10.1038/s41420-022-01048-8)
Supplement: Supplementary file 2 — supplementary figures [file 41420_2022_1048_MOESM2_ESM.docx]

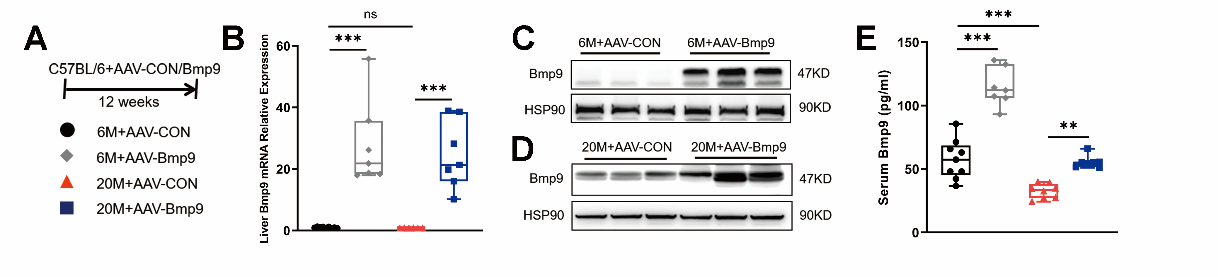


Figure S1. Verification of Bmp9 overexpression in mice after AAV injection through tail vein. (A) 6-month-old and 20-month-old mice were injected with AAV-Bmp9 or AAV-CON, respectively. 12 weeks after injection, mice were euthanized for subsequent experiments. (B) qPCR analysis of Bmp9 mRNA expression in liver. (C,D) WB analysis of protein levels of Bmp9 in liver of 6-month-old mice (C) and 20-month-old (D) mice treated with AAV. (E) Serum level of BMP9 assessed by ELISA. Data presented as mean ± SD. One-way ANOVA was used for comparisons among multiple groups. **P* < 0.05; ***P* < 0.01; ****P* < 0.001. Ns, no significance.

**
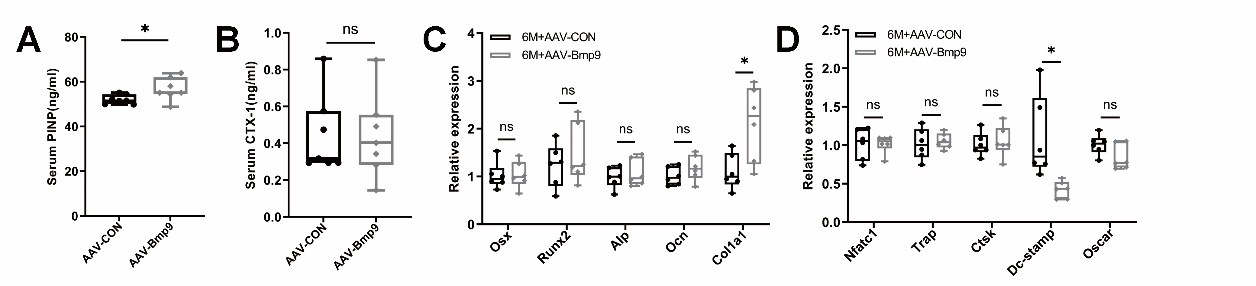
**

Figure S2. Serum levels of bone turnover markers and osteoblast/osteoclast differentiation markers in bone of 6-month-old mice treated with Bmp9 overexpression. (A) Serum level of PINP in 6-month-old mice treated with AAV-CON and AAV-Bmp9. (B) Serum level of CTX-1 in 6-month-old mice treated with AAV-CON and AAV-Bmp9. (C) qPCR analysis of osteoblast differentiation markers in vertebrae. (D) qPCR analysis of osteoclast differentiation markers in vertebrae. Data presented as mean ± SD. A t-test was used for comparison between two groups. **P* < 0.05; ***P* < 0.01; ****P* < 0.001. Ns, no significance.

**
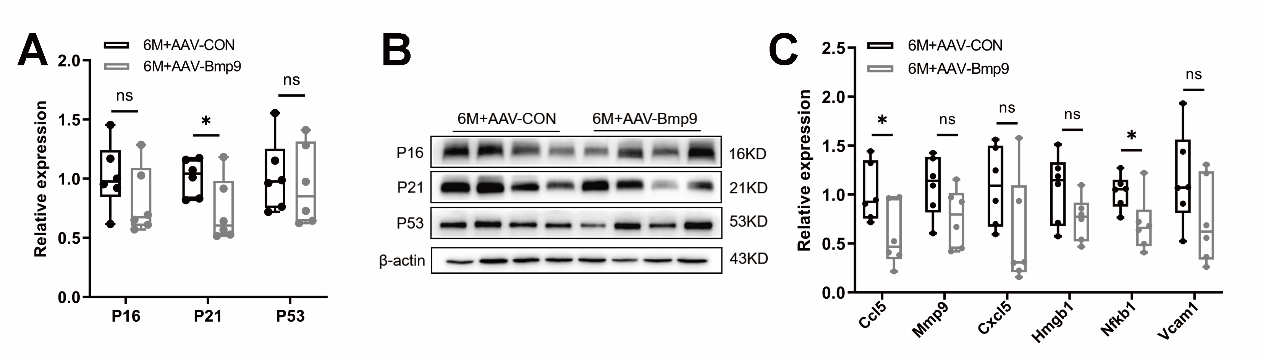
**

Figure S3. Expression of senescent genes and SASPs in bone microenvironment of 6-month old mice treated with Bmp9 overexpression. (A) qPCR analysis of mRNA levels of senescent genes in bone microenvironment. (B) WB analysis of protein levels of senescent genes in bone microenvironment. (C) qPCR analysis of mRNA levels of SASPs in bone microenvironment. Data presented as mean ± SD. A t-test was used for comparison between two groups. **P* < 0.05; ***P* < 0.01; ****P* < 0.001. Ns, no significance.


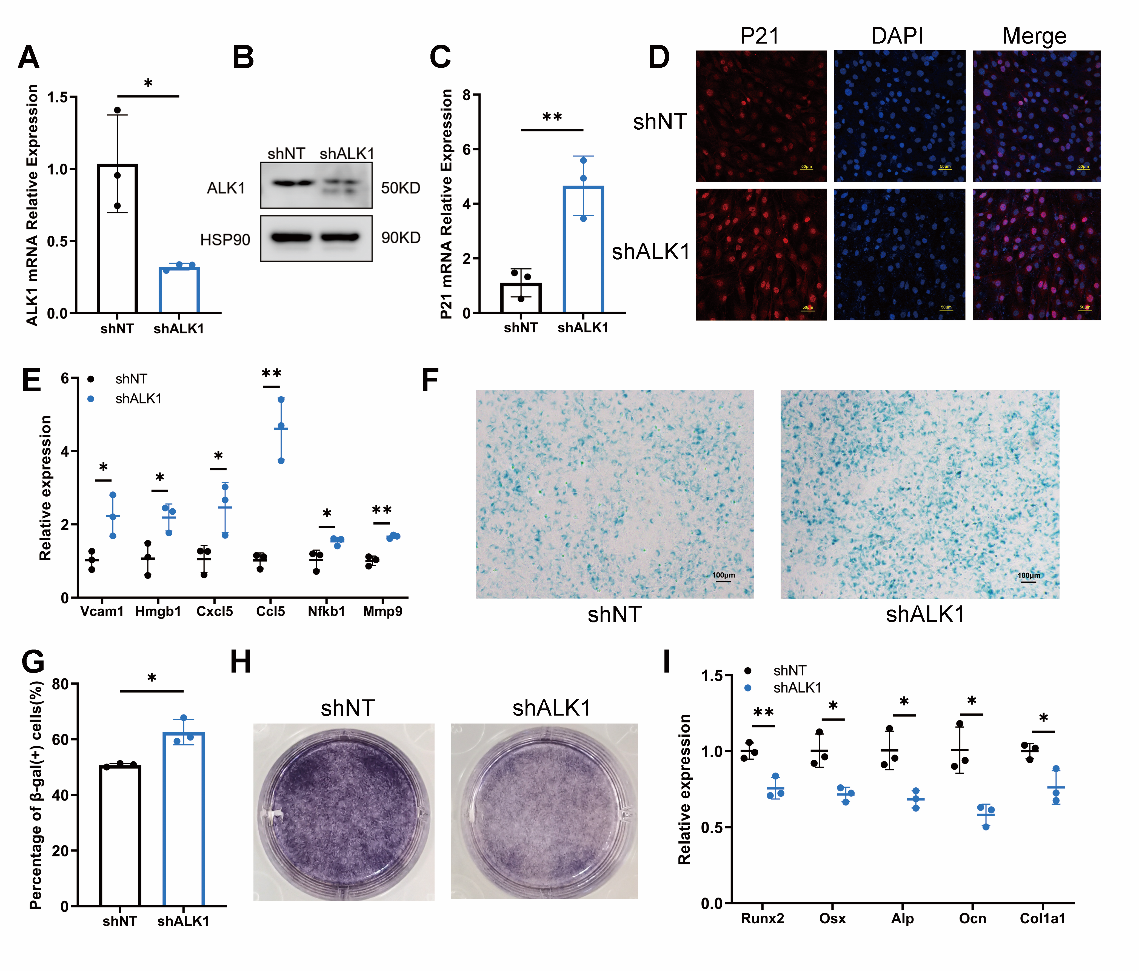


Figure S4. Knockdown of ALK1 accelerates osteoblast senescence induced by H_2_O_2_. (A) qPCR analysis of mRNA levels of ALK1 in control and ALK1 knockdown MC3T3-E1 cells. (B) WB analysis of the protein levels of ALK1 in control and ALK1 knockdown cells. (C) qPCR analysis of mRNA levels of P21 in control and ALK1 knockdown cells after H_2_O_2_ stimulation. (D) Immunofluorescence analysis of the protein levels of P21 in control and ALK1 knockdown cells after H_2_O_2_ stimulation. (Scale bar, 50 μm). (E) qPCR analysis of mRNA levels of SASPs in control and ALK1 knockdown cells after H_2_O_2_ stimulation. (F) β-galactosidase staining of control and ALK1 knockdown cells after H_2_O_2_ stimulation. (Scale bar, 100 μm). (G) The number of β-gal(+) cells was counted for each group. (H) ALP staining of senescent control and ALK1 knockdown MC3T3-E1 cells after osteoblastic induction for 7 days. (I) qPCR analysis of mRNA levels of osteoblastic differentiation markers in senescent control and ALK1 knockdown cells after osteoblastic induction for 7 days. Data presented as mean ± SD. A t-test was used for comparison between two groups. **P* < 0.05; ***P* < 0.01; ****P* < 0.001.


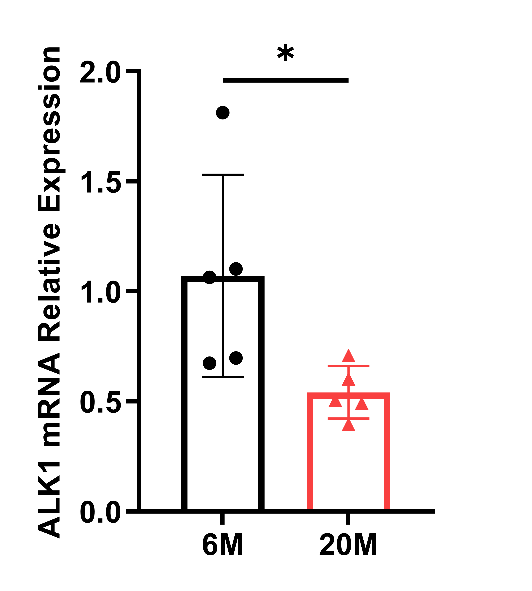


Figure S5. Expression of ALK1 mRNA in 6-month and 20-month old mice, respectively. Data presented as mean ± SD. A t-test was used for comparison between two groups. **P* < 0.05; ***P* < 0.01; ****P* < 0.001.
