# Supplementary material for: BMP9 reduces age-related bone loss in mice by inhibiting osteoblast senescence through Smad1-Stat1-P21 axis
Source: Cell Death Discov. 2022 May 6;8:254. doi: 10.1038/s41420-022-01048-8 (PMC9076651; doi:10.1038/s41420-022-01048-8)
Supplement: Supplementary file 3 — supplementary tables [file 41420_2022_1048_MOESM3_ESM.docx]

Supplementary Table S1. GO analysis of top 10 downregulated biological processes by Bmp9 in senescent osteoblast.

| GO ID | Description | Out | All | p-value | p.adjust |
| --- | --- | --- | --- | --- | --- |
| GO:0035456 | response to interferon-beta | 24 | 62 | 3.45E-24 | 1.91E-20 |
| GO:0035458 | cellular response to interferon-beta | 22 | 51 | 1.54E-23 | 4.26E-20 |
| GO:0006952 | defense response | 96 | 1589 | 2.77E-20 | 5.12E-17 |
| GO:0002376 | immune system process | 128 | 2579 | 7.57E-20 | 1.05E-16 |
| GO:0034097 | response to cytokine | 67 | 927 | 4.72E-18 | 5.23E-15 |
| GO:0009615 | response to virus | 38 | 313 | 8.93E-18 | 8.25E-15 |
| GO:0045087 | innate immune response | 60 | 827 | 2.66E-16 | 2.11E-13 |
| GO:0051607 | defense response to virus | 32 | 245 | 4.49E-16 | 3.11E-13 |
| GO:0006950 | response to stress | 151 | 3691 | 7.67E-16 | 4.72E-13 |
| GO:0034341 | response to interferon-gamma | 25 | 154 | 4.74E-15 | 2.63E-12 |

Supplementary Table S2. Cell lines.

| **Cell line** | **Source and Catalog# or RRID** | **Species, cell type** |
| --- | --- | --- |
| MC3T3-E1 Subclone 4 | ATCC CRL-2593 | Mus musculus; osteoblast |

Supplementary Table S3. Biological Modulators.

| Modulator (protein, small molecule) | Source, Catalog # or RRID | Solvent | Concentration(s) |
| --- | --- | --- | --- |
| β-glycerophosphate disodium salt hydrate | Sigma-Aldrich, G9422 | α-MEM | 10mmol/L |
| Ascorbic acid | Sigma- Aldrich, A92902 | Saline | 50 μmol/L |
| Recombinant mouse BMP9 | Biolegend;553204 | PBS | 100ng/ml |
| 2-NP | Abcam;142704 | DMSO | 50μmol/L |
| LDN193189 | Selleck;s7507 | PBS/saline | 0.5μmol/L(in vitro);3mg/ml(in vivo) |

Supplementary Table S4. Primary antibodies used for western blot and immunofluorescence.

| Primary Antibody | Company | Catalog No. | Application | Dilution | Host species |
| --- | --- | --- | --- | --- | --- |
| Runx2 | CST | 8486 | WB | 1:1000 | Rabbit |
| Osx | Abcam | ab22552 | WB | 1:1000 | Rabbit |
| Hsp90 | Santa Cruz | 13119 | WB | 1:1000 | Mouse |
| β-actin | CST | 4970 | WB | 1:1000 | Rabbit |
| P16 | Abcam | ab211542 | WB | 1:500 | Rabbit |
| P21 | Santa Cruz | 6246 | IF | 1:100 | Mouse |
| P53 | CST | 2524 | WB | 1:1000 | Mouse |
| Stat1 | CST | 14994 | WB  IF | 1:1000  1:400 | Rabbit |
| Smad1 | CST | 9743 | WB | 1:1000 | Rabbit |
| p-smad1/5/9 | CST | 13820 | WB | 1:1000 | Rabbit |
| Bmp9 | Abcam | ab207318 | WB | 1:1000 | Rabbit |
| γ-H2AX | CST | 9718 | IF | 1:400 | Rabbit |
| P21 | CST | 64016 | WB | 1:1000 | Rabbit |
| Alk1 | Abcam | ab263902 | WB | 1:1000 | Rabbit |

Supplementary Table S5. Primer sequences used in this study.

|  | Forehead primer(5'-3') | Reverse primer(5'-3') |
| --- | --- | --- |
| Osx | ATGGCGTCCTCTCTGCTTG | TGAAAGGTCAGCGTATGGCTT |
| Runx2 | TTCAACGATCTGAGATTTGTGGG | GGATGAGGAATGCGCCCTA |
| Alp | CCAACTCTTTTGTGCCAGAGA | GGCTACATTGGTGTTGAGCTTTT |
| Ocn | AAGGTAGTGAACAGACTCCGGC | CTCGTCACAAGCAGGGTTAAGC |
| Col1a1 | GCTCCTCTTAGGGGCCACT | CCACGTCTCACCATTGGGG |
| Nfatc1 | GACCCGGAGTTCGACTTCG | TGACACTAGGGGACACATAACTG |
| Trap | CACTCCCACCCTGAGATTTGT | CATCGTCTGCACGGTTCTG |
| Ctsk | GAAGAAGACTCACCAGAAGCAG | TCCAGGTTATGGGCAGAGATT |
| Dc-stamp | TACGTGGAGAGAAGCAAGGAA | ACACTGAGACGTGGTTTAGGAAT |
| Oscar | TACCGCAAGACAGACTGGG | GGGAGCTGATCCGTTACCA |
| P16 | CGCAGGTTCTTGGTCACTGT | TGTTCACGAAAGCCAGAGCG |
| P21 | CCTGGTGATGTCCGACCTG | CCATGAGCGCATCGCAATC |
| P53 | GCGTAAACGCTTCGAGATGTT | TTTTTATGGCGGGAAGTAGACTG |
| Mmp9 | CTGGACAGCCAGACACTAAAG | CTCGCGGCAAGTCTTCAGAG |
| Hmgb1 | GGCGAGCATCCTGGCTTATC | GGCTGCTTGTCATCTGCTG |
| Nfkb1 | ATGGCAGACGATGATCCCTAC | TGTTGACAGTGGTATTTCTGGTG |
| Vcam1 | AGTTGGGGATTCGGTTGTTCT | CCCCTCATTCCTTACCACCC |
| Ccl5 | GCTGCTTTGCCTACCTCTCC | TCGAGTGACAAACACGACTGC |
| Cxcl5 | TCCAGCTCGCCATTCATGC | TTGCGGCTATGACTGAGGAAG |
| Bmp9 | CAGAACTGGGAACAAGCATCC | GCCGCTGAGGTTTAGGCTG |
| Stat1 | TCACAGTGGTTCGAGCTTCAG | GCAAACGAGACATCATAGGCA |
| Smad1 | GCTTCGTGAAGGGTTGGGG | CGGATGAAATAGGATTGTGGGG |
| Smad5 | TTGTTCAGAGTAGGAACTGCAAC | GAAGCTGAGCAAACTCCTGAT |
| Smad9 | CGGGTCAGCCTAGCAAGTG | GAGCCGAACGGGAACTCAC |
| Alk1 | CTGGGTGCTCTAGGCTTGTG | GCCCGTAGTACAGTCGCTG |
| β-actin | GGCTGTATTCCCCTCCATCG | CCAGTTGGTAACAATGCCATGT |
