# Supplementary figures and images for: BMP9 reduces age-related bone loss in mice by inhibiting osteoblast senescence through Smad1-Stat1-P21 axis
Source: Cell Death Discov. 2022 May 6;8:254. doi: 10.1038/s41420-022-01048-8 (PMC9076651; doi:10.1038/s41420-022-01048-8)

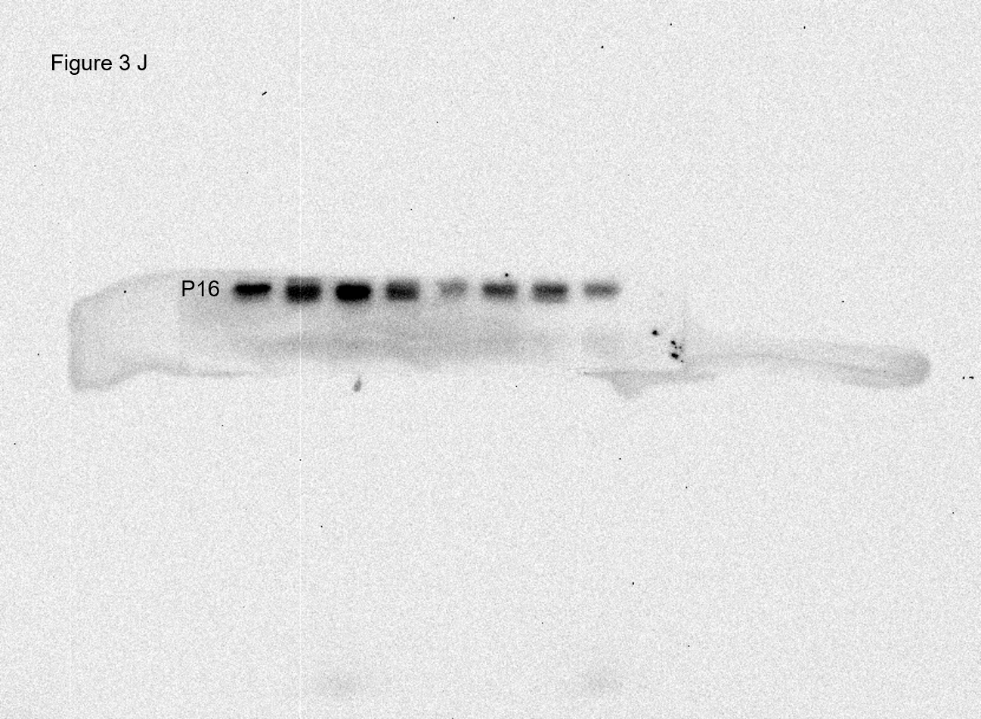

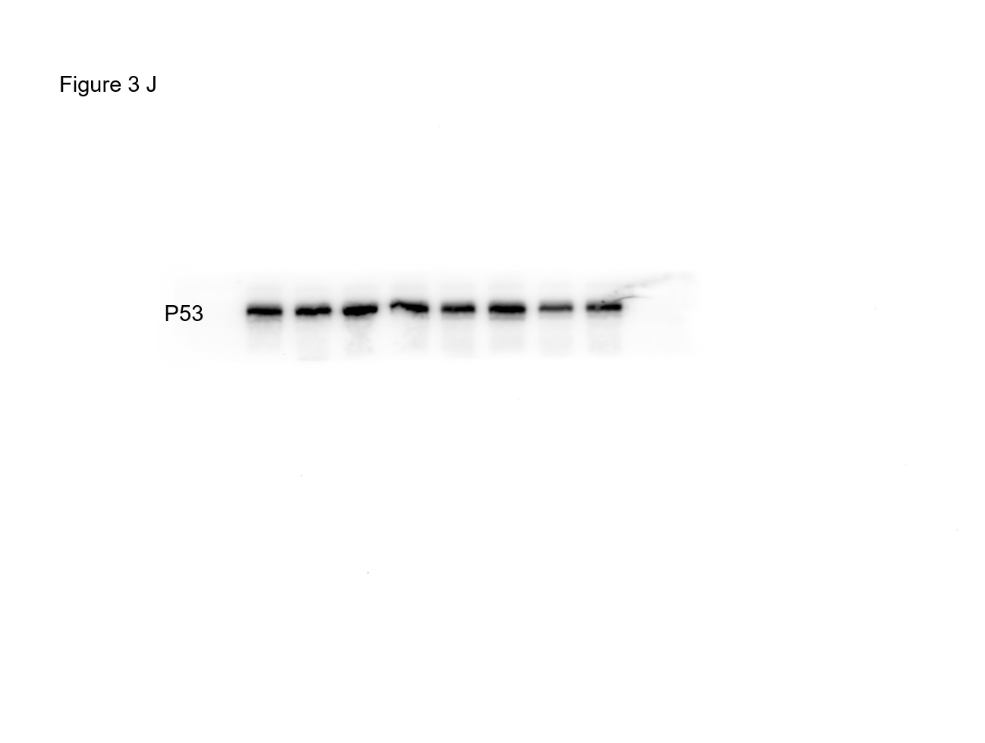

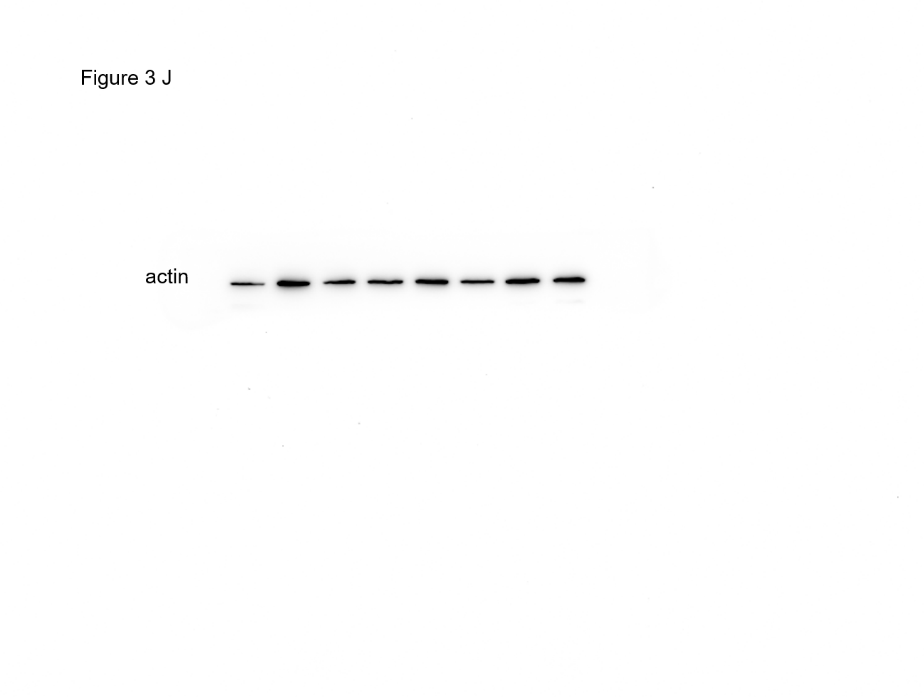

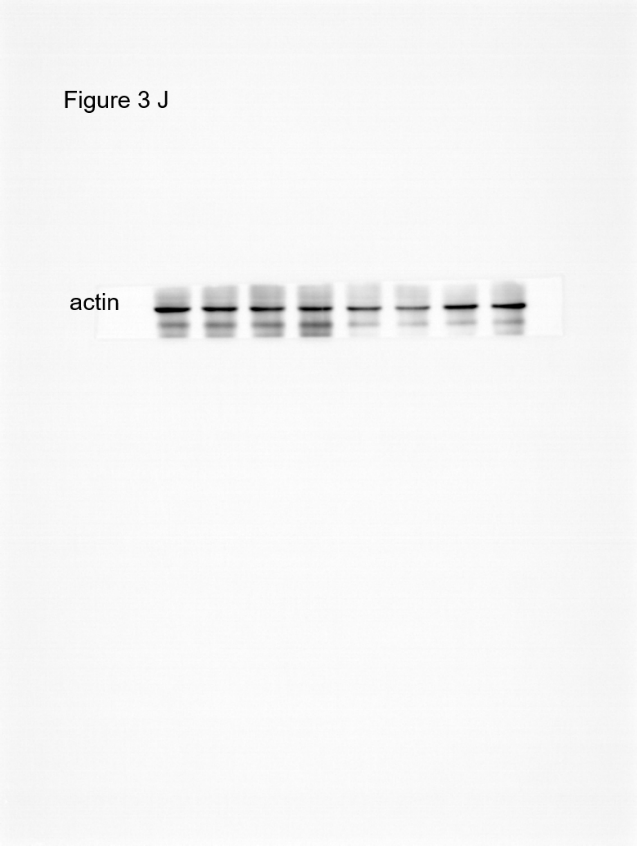

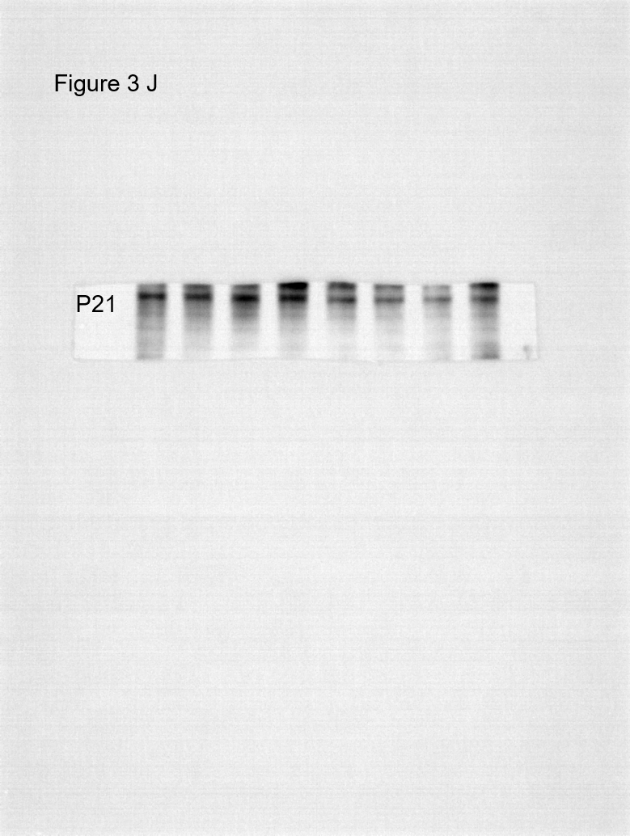

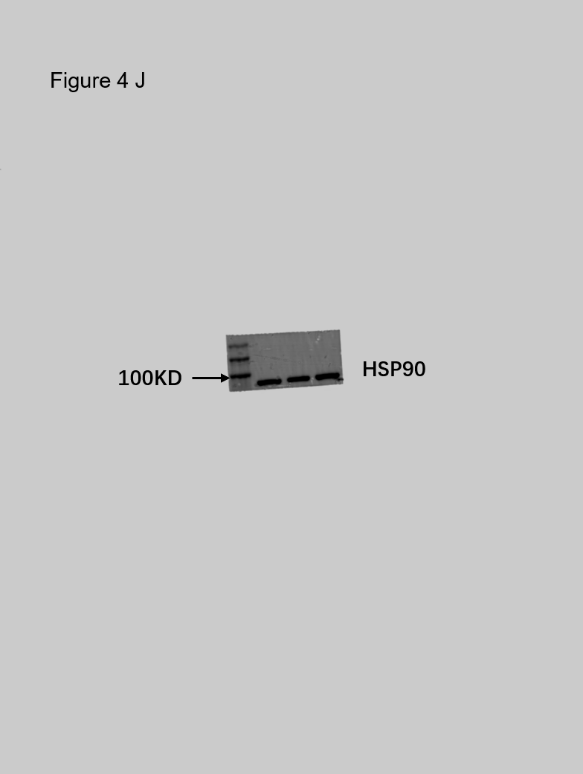

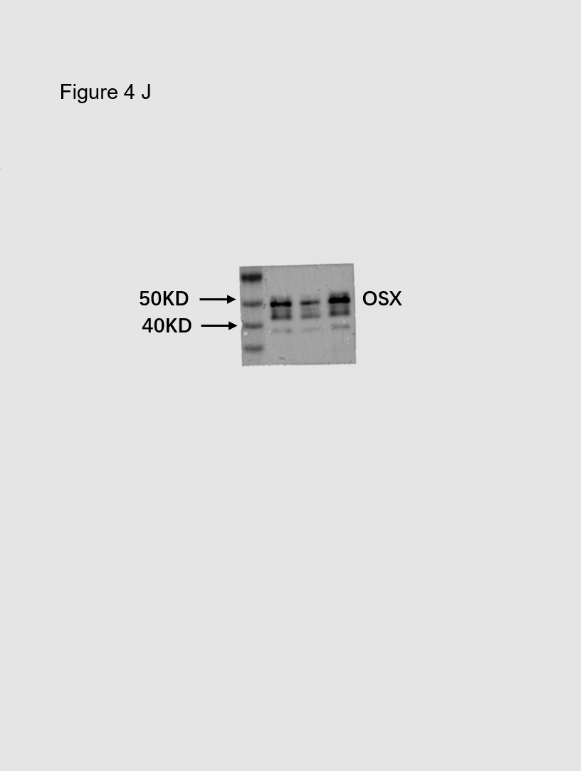

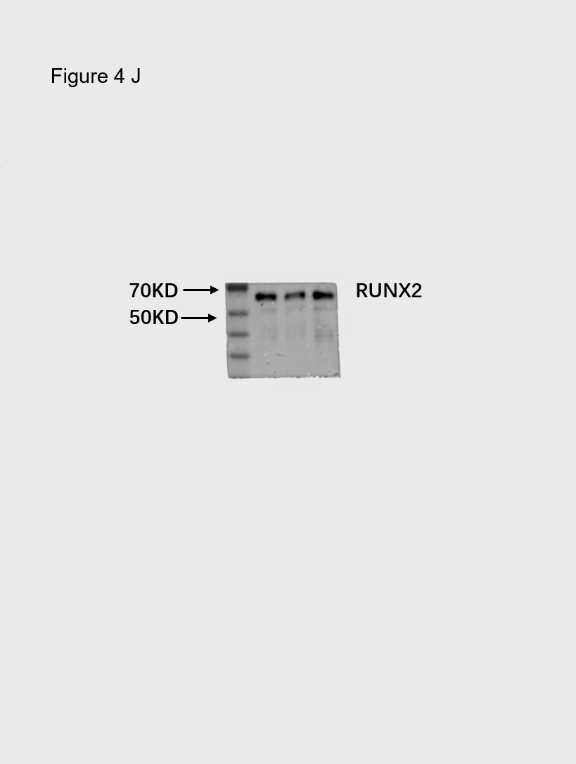

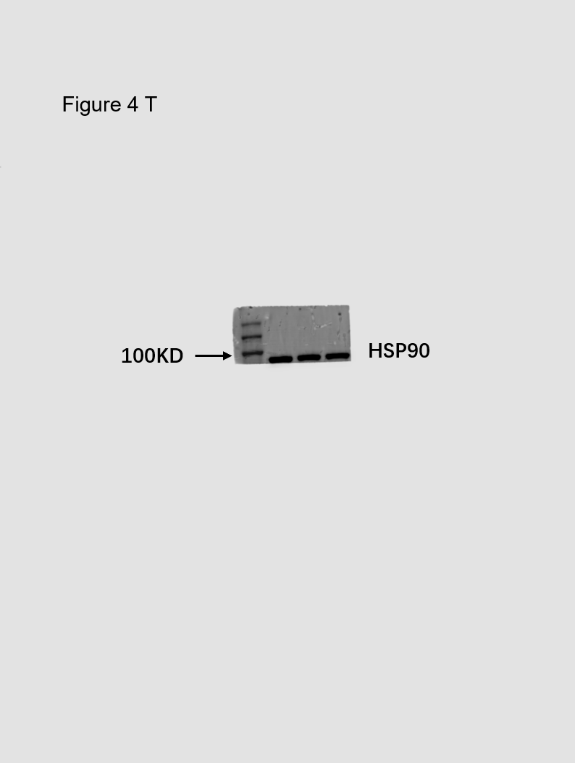

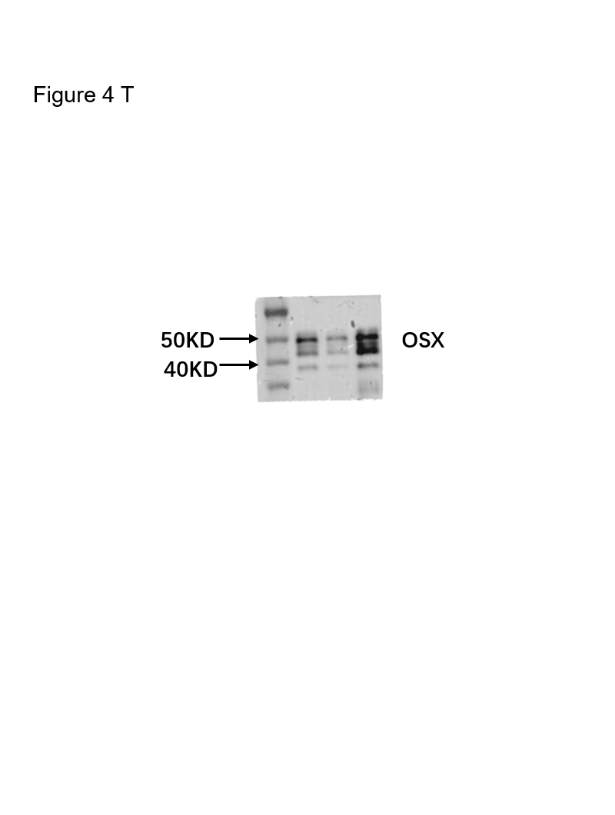

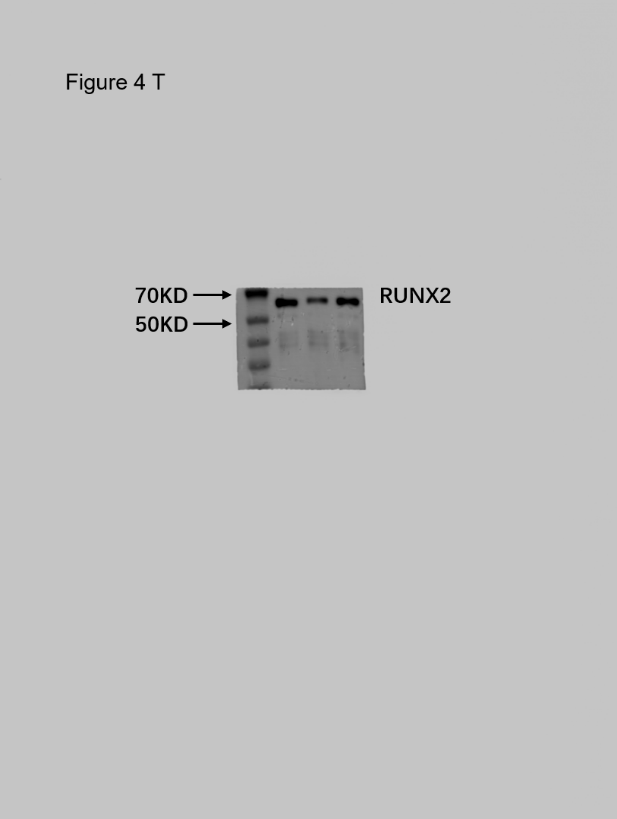

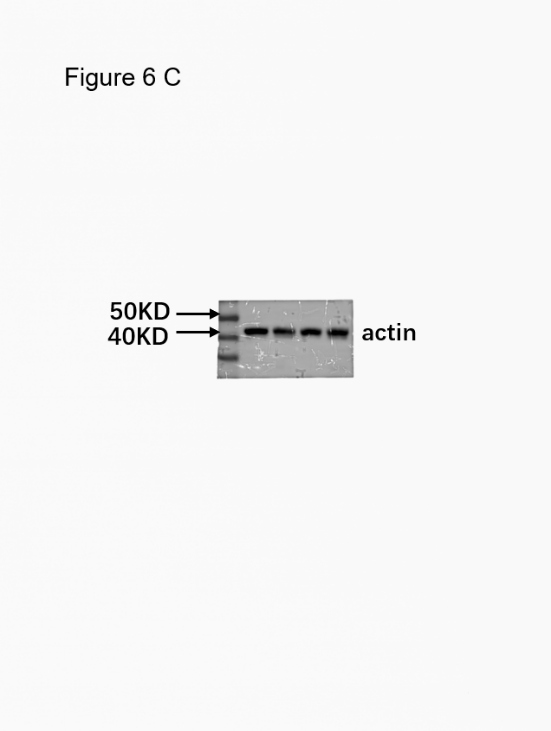

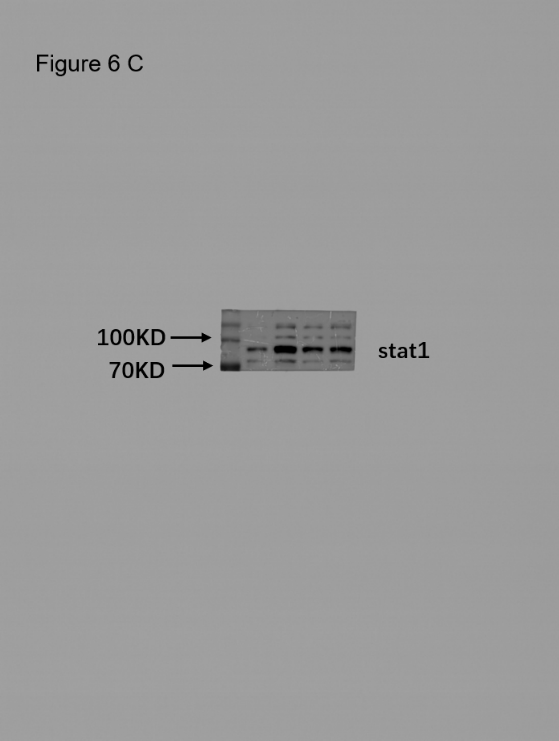


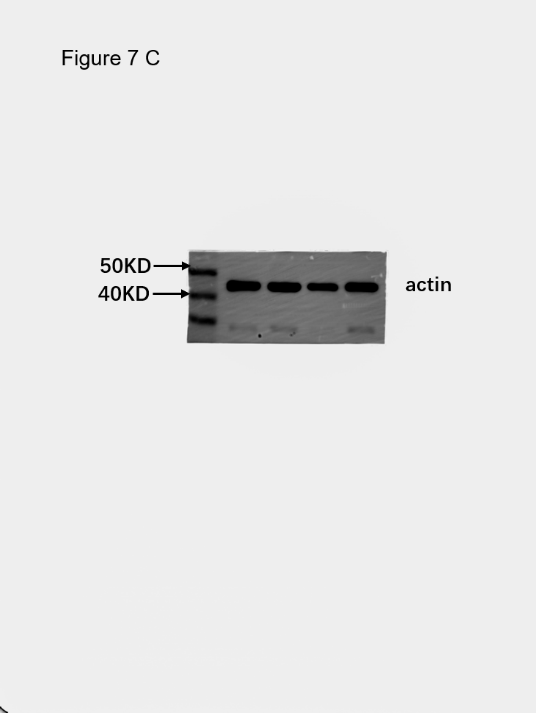

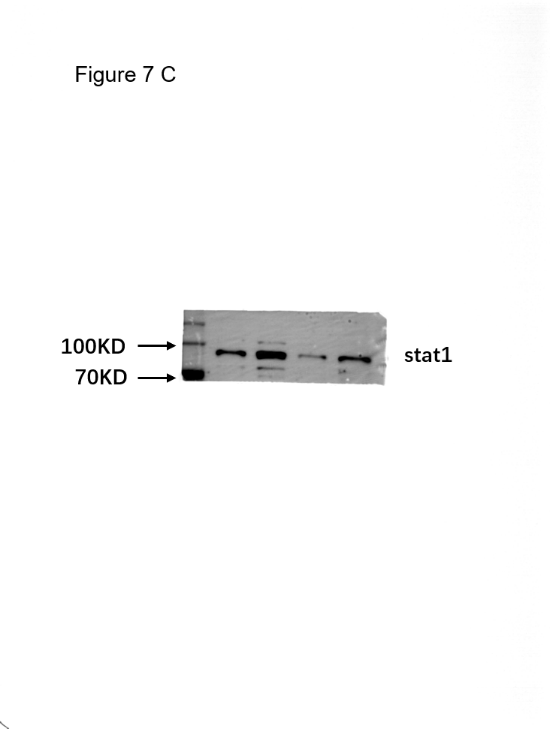

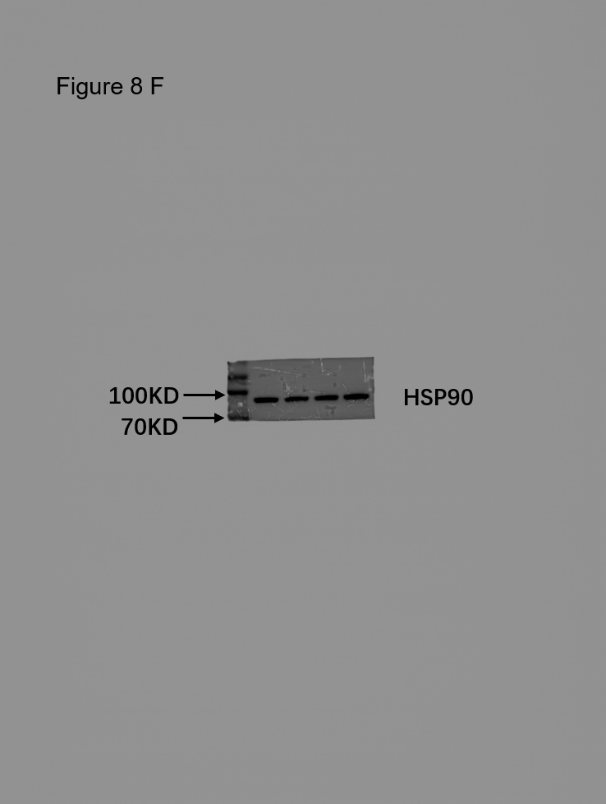

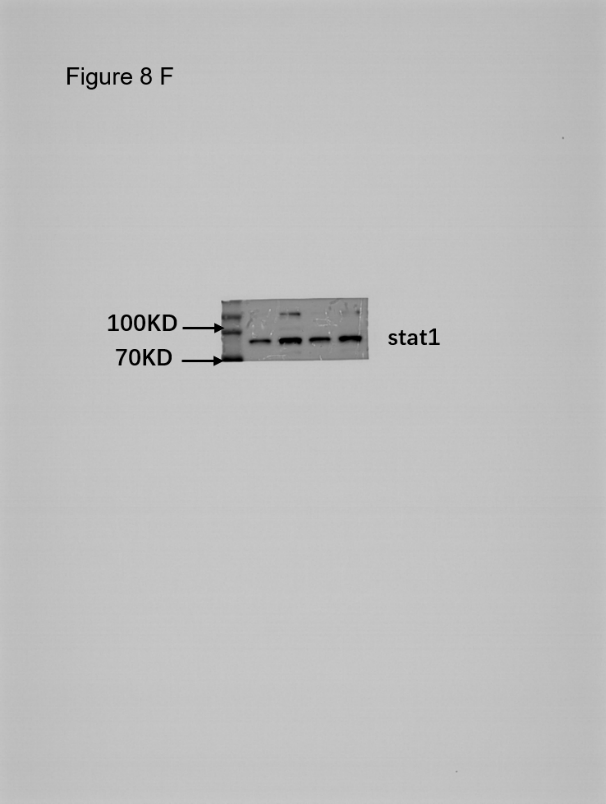


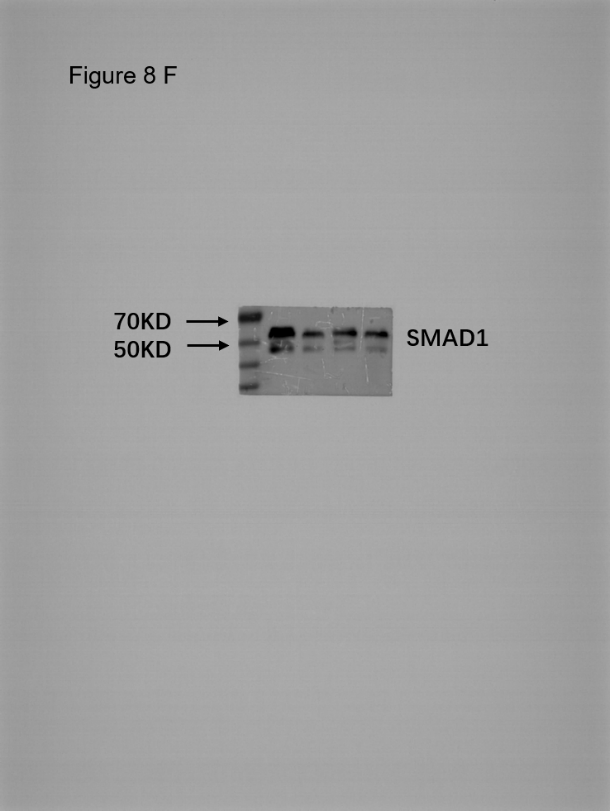

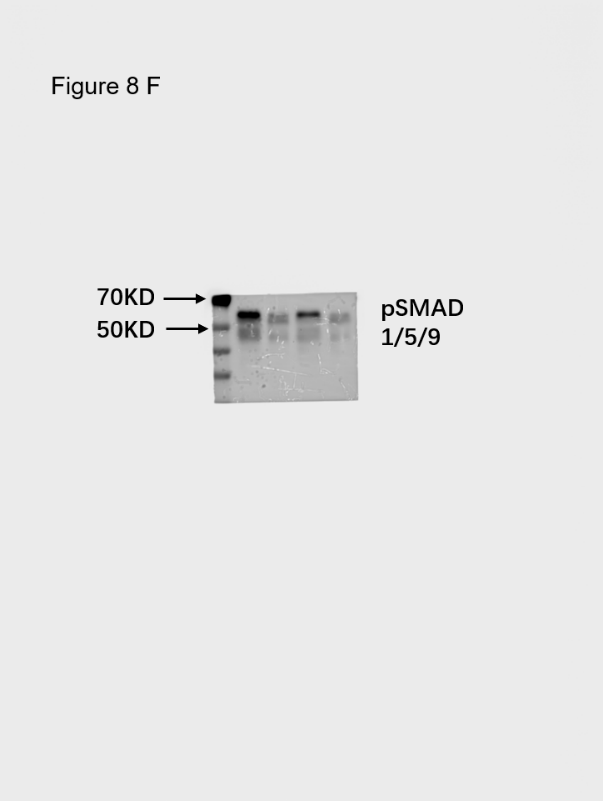

Supplement: Supplementary file 4 — original WB [file 41420_2022_1048_MOESM4_ESM.docx]
